# Impact of lifting school mask mandates on community SARS-CoV-2 cases, hospitalizations, and deaths: a retrospective observational study

**DOI:** 10.3389/fpubh.2025.1579202

**Published:** 2025-06-12

**Authors:** Zeynep Ertem, Anseh Danesharasteh, Sonia T. Anand, Nicholas J. Jackson, Richard E. Nelson, Elissa M. Schechter-Perkins, Lloyd Fisher, Shira Doron, Westyn Branch-Elliman

**Affiliations:** ^1^School of Systems Science and Industrial Engineering, Binghamton University, State University of New York, Binghamton, NY, United States; ^2^VA Boston Cooperative Studies Program, Boston, MA, United States; ^3^UCLA David Geffen School of Medicine, Los Angeles, CA, United States; ^4^IDEAS Center, Veterans Affairs Salt Lake City Healthcare System, Salt Lake City, UT, United States; ^5^Department of Internal Medicine, University of Utah School of Medicine, Salt Lake City, UT, United States; ^6^Department of Emergency Medicine, Boston University School Chobanian and Avedisian of Medicine and Boston Medical Center, Boston, MA, United States; ^7^Reliant Medical Group, Worcester, MA, United States; ^8^Department of Pediatrics, UMass Medical School, Worcester, MA, United States; ^9^Division of Infectious Diseases and Geographic Medicine, Tufts Medical Center, Boston, MA, United States; ^10^Greater Los Angeles VA Medical Center, Los Angeles, CA, United States; ^11^VA Center for the Study of Healthcare Innovation, Implementation, and Policy (CSHIIP), Los Angeles, CA, United States

**Keywords:** COVID-19, schools, mask, infection prevention, respiratory virus, SARS-CoV-2, healthcare policies

## Abstract

**Background:**

School masking mandates were widely adopted as a pandemic control measure, however, limited data are available regarding their effectiveness as a strategy for reducing burden of disease in the surrounding community.

**Objective:**

To evaluate the impact of school masking policy de-adoption (mask-lifting) on SARS-CoV-2 incidence rates, hospitalizations, and deaths in the surrounding community.

**Methods:**

*Design*: Retrospective observational study with an event study design, a difference-in-difference method; a target trial emulation (TTE) framework was applied as a secondary analysis. *Cohort creation*: Data collected from 9/2021 to 6/2022 on SARS-CoV-2 cases, hospitalizations, deaths and vaccination rates were combined with district-level masking policy data. *Analysis*: In the event study, the impact of masking policy de-adoption on SARS-CoV-2 cases per 100,000 county residents stratified by age during the 8-week period following the policy change was estimated. Effects on hospitalization and deaths per 1,000,000 residents were secondarily estimated. In a secondary analysis, a target trial emulation framework was applied to estimate average treatment effects.

**Results:**

*N* = 3,970 districts composed of 53,453 schools were included in the cohort. In the event study, no consistent trends for COVID-19 case rates were identified for the whole cohort or for any age group. For the whole cohort, there was a statistically significant increase found 6–8 weeks following the policy change (maximum increase, 1.91 hospitalizations per 1,000,000 county residents); increases in hospitalizations were also found in the stratified analysis for all age groups, although absolute impacts were small. An increase in deaths was found during the period from 4 to 7 weeks following the policy change (maximum increase 0.62 deaths per 1,000,000 residents). In the stratified analysis, small increases in death rates were seen in 50–69 year olds (range, 0.088–1.49) and >70 year olds (range, 0.23–2.58) but not in younger groups. In the TTE framework, cases, hospitalizations, and deaths were similar in control and intervention counties.

**Conclusion:**

This study evaluating the impact of lifting of mask mandates in schools, analyzed in two ways, was consistent results ranging from no impact to a small but statistically significant impact of the policy change on SARS-CoV-2 case and severe outcomes rates in the surrounding community. Findings can be used to inform future pandemic policy responses for elementary and secondary schools.

## Background

**Figure 1:**
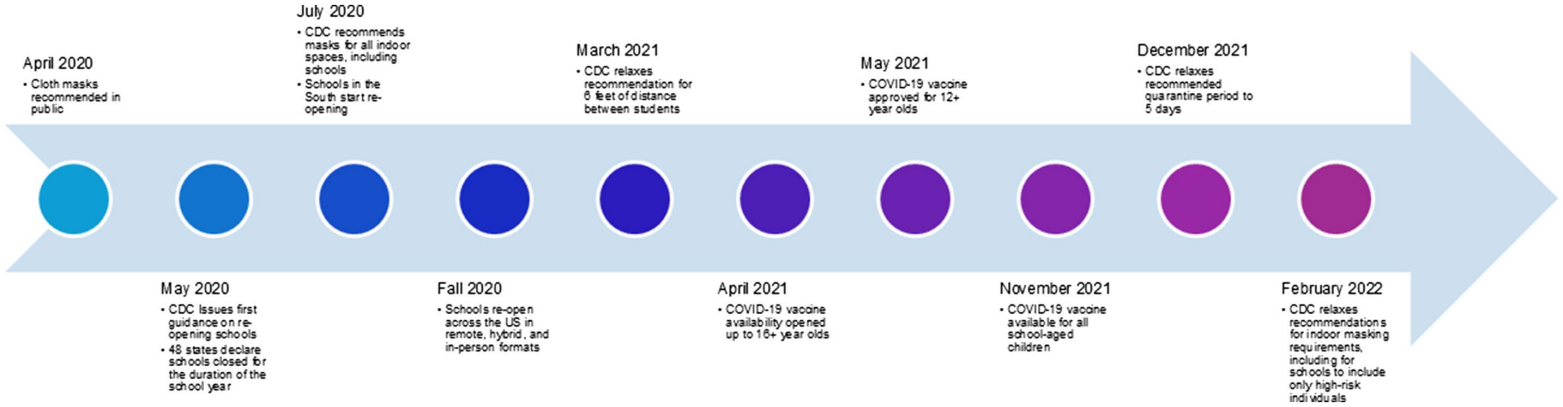
A timeline of key US Centers for Disease Control and Prevention policy recommendations and other critical milestones. Timeline created based on information gathered by Education Week and the Boston Herald (6, 7).

In March 2020, in the setting of a public health emergency, schools were closed to limit the spread of SARS-CoV-2. Modeling studies developed prior to the COVID-19 pandemic suggested that transmission in schools is a major driver of respiratory viral spread in the community (1–4). Based on these extrapolated data and US Centers for Disease Control and Prevention (CDC) recommendations, as schools reopened, non-pharmaceutical interventions including mask mandate policies were implemented in school settings to reduce the number of cases among students and staff (5) and surrounding communities. A timeline of major CDC recommendations about in-school mitigation and other critical developments is presented in Figure 1 (6, 7).

During the intervening period since the initial recommendation for universal masking, multiple studies examined the impacts of different masking policies at the community-level on SARS-CoV-2 spread; observational data have generally found a short-lived, condition-dependent, small but measurable reduction in case rates in counties that adopted more restrictive masking policies (8–10).

The impact of in-school masking policies on case rates in the schools themselves has also been examined in several studies; results of these studies are mixed, with some suggesting a 30% decrease in cases among students and staff and others suggesting limited or no effect (11, 12); these studies are likely affected by a variety of confounders and impacts of in-school masking policy on cases in schools remains uncertain.

**Figure 2:**
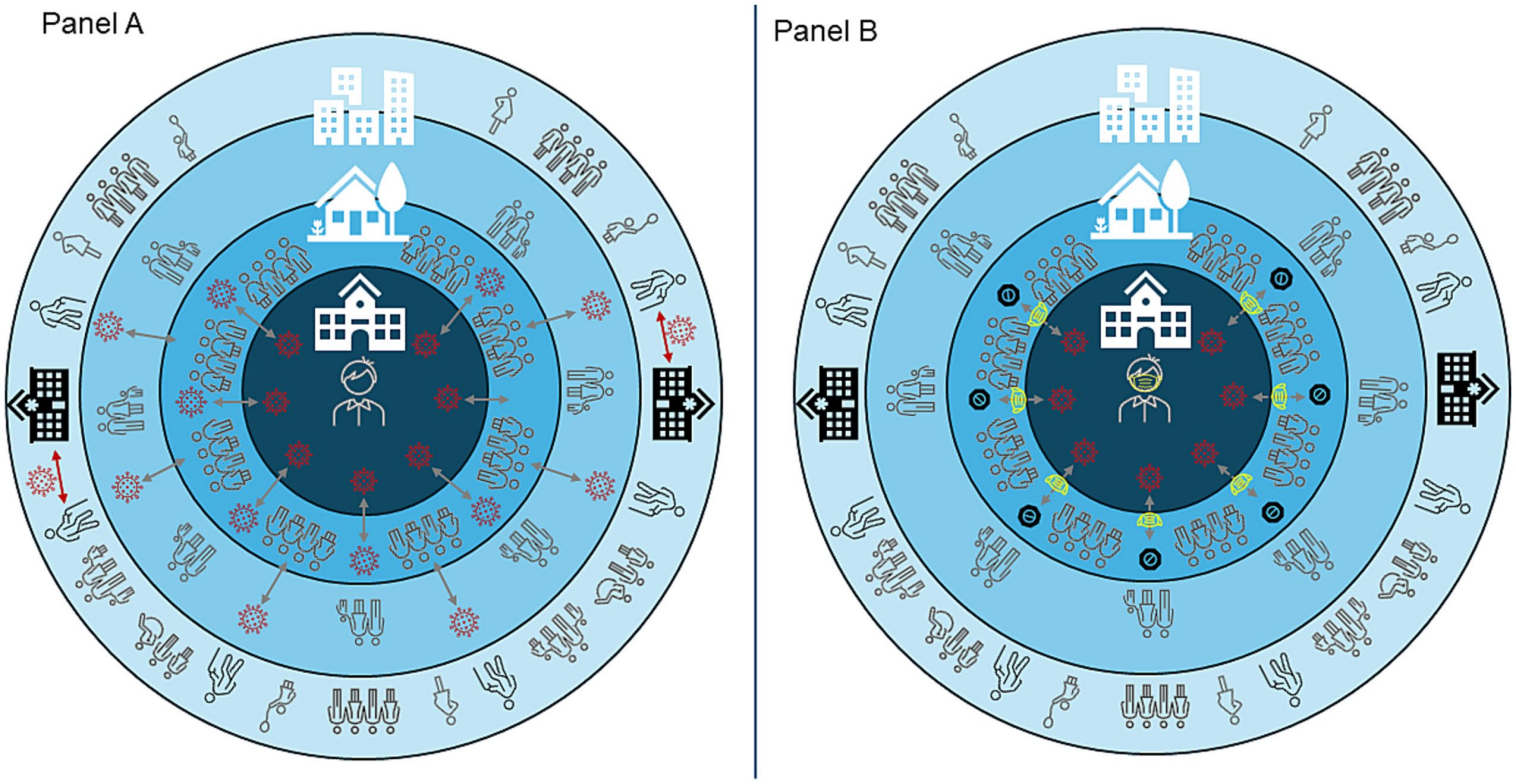
A theoretical model of the role schools play in SARS-CoV-2 spread in the community and the hypothetical impact of masks on reducing spread. Panel A depicts in-school contacts hypothetically leading to spread within the home which then leads to spread within the community and attributable hospitalizations among older adults at higher risk of severe COVID-19. Panel B depicts the hypothetical impact of in-school masking policies on the community. In this scenario, masking prevents transmission of SARS-CoV-2 in schools and this reduction leads to lower in-home and community transmission. When community transmission is lowered via mitigation in schools, severe outcomes among older adults are prevented.

Prior modeling work has suggested that children attending school have more contacts than adults and that these in-school contacts lead to more transmission opportunities that then lead to spread in adults and in the community at-large (Figure 2) (2). Among those infected with SARS-CoV-2, increasing age is strongly correlated with increased risk of severe disease; primary and secondary school aged students are generally at low-risk of severe outcomes (13). Thus, a major concern driving in-school masking policy recommendations was that children would become infected and then spread the disease to their adult contacts, who are at higher risk of symptomatic infection and severe outcomes. This onward spread could then continue to high-risk members of the community, contributing to increases in COVID-19 hospitalizations and deaths. One study conducted during the early phase of the pandemic, before vaccines were available for adults, suggested masking requirements in schools reduced community spread (14) and deaths. However, despite these theoretical concerns and data generated during the early phases of the pandemic, there are limited real-world data about the impact of interventions implemented in school settings on community spread of SARS-CoV-2, particularly following widespread availability of vaccination and immunity from infection, both of which reduce severe disease (15).

In the United States, schools are under local control; although the CDC is able to make recommendations about mitigation of in-school transmission, the national public health agency does not dictate local policy. As a result, in-school mitigation measures varied widely. The aim of this nationwide retrospective observational cohort study (16, 17) was to leverage this variation to estimate the impact of lifting mask requirements in schools on incidence rates of SARS-CoV-2 in the surrounding community. SARS-CoV-2 related community hospitalization and death rates, stratified by age, were evaluated secondarily. The primary hypothesis was that weekly incidence rates of SARS-CoV-2 infections would be higher in counties that de-adopted in-school masking policies versus those that maintained them. The secondary hypothesis was that hospitalizations and deaths would be higher in these counties.

## Methods

A retrospective, national cohort with longitudinal data regarding in-school mitigation measures, district demographic variables, and SARS-CoV-2 outcomes and vaccination rates at the county-week level was created by combining different datasets (Supplementary Table 1). County FIPS codes were used to link the different data elements to create the full cohort. Data were analyzed using an event study framework which included all counties with available information about policy and using a target trial emulation framework.

### Data sources

*In-school mitigation measures*: District-level masking policy data were obtained from the Burbio dataset, which includes a national sample of public school districts across the US. Districts included in the Burbio dataset underwent weekly review by trained human reviewers of in-school non-pharmaceutical mitigation measures, including in-school masking, testing, and vaccination policies (18, 19). Data were collected manually from publicly available sources, such as district websites and local news sources. Counties in the Burbio dataset are selected to be representative of US counties as a whole and 70% of all US elementary and secondary students nationwide are represented. In-school masking policies in the Burbio dataset were recorded as present, absent, or partial (which included optional participation). Additional school mitigation policies included in the Burbio and our final dataset included vaccination policies (categorized as all eligible populations, limited to high school populations, and none/selected populations [e.g., for athletes and extracurricular activities only]) and asymptomatic testing policies (categorized as universal [e.g., all students required to participate], partial [e.g., optional/opt-in, metric-dependent, variation by grade level], selected populations only [e.g., limited to participation in sports or extracurriculars], required for specific ages only [typically pre-kindergarten and/or kindergarten], or none [e.g., none, limited information, at-home testing only]).

*District demographic data*: Data about individual school districts and the number of schools within each district were obtained from the National Center for Education Statistics (20).

*Community activity*: Data on community mobility were obtained from Google COVID-19 Community Mobility Report, which tracks community activity levels, including recreational activities (21).

*SARS-CoV-2 incidence, hospitalizations, and deaths*: Community incidence rates and hospitalizations were obtained from the CDC restricted dataset, which includes weekly county-level data about SARS-CoV-2 incidence rates, hospitalizations, and deaths (22). Data are stratified into age categories by decade of age (0–9, 10–19, 20–49, 50–69, 70+). Data on vaccination rates by age were also obtained from the CDC (20).

*Community risk-level*: Community COVID-19 risk level was obtained from COVID Act Now (23). The community COVID-19 risk level is a combined measure of weekly incident SARS-CoV-2 cases per 100,000 county residents, weekly COVID-19 hospital admissions per 100,000 county residents, and the percentage of staffed inpatient beds occupied by COVID patients. Data underling the community risk score for COVID Act Now were obtained from CDC’s Community Level framework.

### County inclusion criteria

Our primary dataset was from Burbio, which includes school-level non-pharmaceutical interventions such as masking policy status (on/off), COVID-19 testing, and vaccination programs. Masking policy status was categorized as “Fully required,” “Partially required,” and “Not required.” Additional data sources were then linked to the Burbio data using county FIPS code and date, including COVID-19 cases, hospitalizations, deaths, age-stratified vaccination rates, demographics, poverty levels, urban/rural classifications, and google mobility trends. Counties with missing data were excluded.

For the event study, a “change” was defined as relaxation of the masking policy, which encompassed switching from “fully required” to either partially or not required, or partially required to not required. Districts that switched their policy within 8 weeks of the initial change were also excluded. For the target trial emulation framework approach, districts with “partially required” were excluded.

### Outcomes and exposures

*Outcome*: The primary outcome was county-level incidence of SARS-CoV-2 during the 8-week period following the policy change; hospitalizations, deaths, and all outcomes stratified by age were evaluated secondarily. Given the nature of the school calendar, trends 4 weeks prior the policy change and 8 weeks after the policy change were evaluated. To maintain patient confidentiality, the CDC datasets provide information about age stratified by decade of life. Thus, age categories considered in the analysis included 0–9 (e.g., elementary-school aged populations), 10–19 (e.g., middle and high school aged populations), 20–69 year olds (adults), and >70 year olds (older adults). By necessity, the 0–9 year old group included individuals too young to attend elementary schools, because a breakdown of cases, hospitalizations, and deaths by 0–4 and 5–9 was not available. Due to the nature of individuals greater than 70 being at particularly high-risk of severe COVID-19 outcomes, there is a focus on this segment of the population.

*Exposure*: The primary exposure of interest was the relaxation of the in-school masking requirement; the date of lifting was defined as the time the Burbio dataset changed the district from “present” or “partial” to “absent” masking policies.

#### Analysis

##### Event study framework

An event study framework, a causal inference design that leverages natural variation in timing of policy changes, was estimated to evaluate the primary study aim: the impact of in-school masking policy de-adoption on incidence of COVID-19 cases, hospitalizations, and deaths in the surrounding community. Event study frameworks are an extension of the difference-in-difference approach that allows for lags and leads of the policy change being evaluated while simultaneously accounting for fixed effects that are impacted by a specific county of interest and by variation in calendar time (24). This analytic strategy was selected because the impact of policy changes is not likely to be immediate, but rather only manifest after a period of time. Given the nature of COVID-19 spread, case rates are not expected to rise immediately after a policy change. Similarly, hospitalization and death rates are expected to occur after a longer time interval following the policy switch as there is a delay between the time of the infection and severe outcomes.

The analysis evaluating COVID-19 case rates was completed with a 1-week lag, to account for the time between an in-school transmission event and secondary cases in the community while a 2-week lag was used for hospitalizations and deaths to account for the time between infection and severe outcomes.

##### Data analysis: event study

We employed an event study framework to assess our main objective: examining the impact of changes in in-school masking policies on COVID-19 incidence in the surrounding community, stratified by age group. The analysis focused on COVID-19 cases with a 1-week lag as the main outcome, while COVID-19 hospitalizations and deaths with a 2-week lag were estimated secondarily. The event study framework is represented by Equation 1. All analyses were conducted using Python and STATA 17.


(1) $$y_{\mathrm{st}}=\alpha +\sum_{j=2}^{J}\beta_{j}{\left(\mathrm{lag}\;j\right)}_{\mathrm{st}}+\sum_{k=1}^{K}\gamma_{k}{\left(\text{lead}\;k\right)}_{\mathrm{st}}+\mu_{s}+\lambda_{t}+X_{\mathrm{st}}\Gamma +\varepsilon_{\mathrm{st}}$$

Where y_st_ is our outcome of interest for county “s” at time “t.” μ_s_, and λ_t_ represent county and time fixed effects, respectively, and X_st_ refers to the time-varying control variable.

All outcomes were adjusted for county-level vaccination, demographics, and poverty rates and school-level student testing and vaccination programs. A *p*-value <0.05 was used to determine statistical significance. To ensure robustness of findings, sensitivity analyses with 0 and 2-week lags for cases only were also estimated.

### Observational cohort with a target trial emulation framework approach

Target trial emulation (TTE) is a causal-inference approach to analyzing observational data that emulates a hypothetical randomized control trial. TTE is used when randomized control trials are generally considered to be not feasible, practical or ethical. Although designed to measure direct impacts, the TTE framework can be applied to minimize biases from poor study design and account for time. Thus, aligned with the standards of this approach, we adopted most of the design principles from randomized trials to emulate a trial to answer our causal question of estimating the effect of lifting mask mandates in schools on SARS-CoV-2 incidence rates in the community. A summary of the protocol for the TTE framework evaluation is presented in Table 1.

**Table 1:** Target trial framework design and emulation strategy.

Protocol component	Target trial	Emulation
Eligibility criteria	Enrollment period: 08/22/21–06/26/2022 Public school district across the US Universal masking policy before 10/28/2021 Age groups: 0–9, 10–19, 20–49, 50–69, 70+ Whole population is eligible	Same, with the following additions: US counties with one school district and in-school masking policy Data on in-school masking -policy available
Treatment strategies	Intervention: Lifting of in-school universal masking requirement in the week of 10/28/2021 Control: Maintenance of in-school universal masking requirement	Same
Assignment procedures	Counties are randomized to one of the treatment strategies	Counties assigned to each treatment strategy are assumed to be comparable through the use of inverse probability weighting
Follow-up period	Time zero at 10/28/2021 Study ends at 8 weeks after 10/28/2021 (study end: 1/6/2022)	Same. Censoring will occur when there is switching of treatment strategies or data becomes unavailable.
Outcome	Primary: county-level incidence of COVID-19 per 100,000 county residents during the follow-up period Secondary: county-level COVID-19 hospitalizations and deaths per 100,000 county residents during the follow-up period All outcomes stratified by decade of life	Same
Causal contrasts of interest	Intention-to-treat effect Per-protocol effect	Per-protocol effect
Analyses	Intention-to-treat analysis: estimate the outcome rates in each group with adjustment for baseline confounders using GEE models based on their initial treatment assignment. Per-protocol analysis: same as intention-to-treat analysis with censoring at non-adherence and adjusting for predictors of both adherence and the outcome.	Same as the per-protocol analysis using IP weighting to adjusting for potential time-varying confounding and selection bias due to loss to follow-up.

*Eligibility criteria*: Public school districts across the United States with a universal masking policy in effect prior to 10/28/2021 were potentially included. Counties eligible for inclusion in the study were those with one school district for the entire county and data about the district-level masking policy available. Only counties with one school district were included because outcome data were only available at the county-level. To avoid mixed and partial policy effects caused by different school districts with potentially different masking policies, counties with multiple school districts were excluded. The study start date was selected because it was a common date of school masking de-adoption across the country and selection of one single date of masking policy change allows for control of seasonal impacts and transmission patterns. Counties with more than one district per county and that did not have a masking requirement before 10/28/2021 were excluded (please see Supplementary materials for additional details).

*Treatment strategies*: The intervention of interest was lifting of the in-school universal masking requirement within a public school district. Those districts that met all requirements were considered the intervention arm. Control counties/districts were those that maintained a masking mandate after 10/28/2021.

*Assignment procedures*: Counties assigned to each treatment strategy are assumed to be comparable through the use of inverse probability weighting. We assume randomization conditional on the following baseline covariates: U. S. region, urban/rural, vaccination rate, risk level, google mobility trends, in-school student testing and student vaccination program.

*Follow up period*: The follow-up period was the 8 weeks following the masking policy change (e.g., 10/28/2021–01/06/2022), which included both the Delta period and part of the Omicron BA.1 surge. An 8-week follow up period was selected to account for school vacation, which typically starts the last week of December.

*Causal contrasts of interests*: The observational data were analyzed using a per-protocol approach. Districts that changed their masking policy (i.e., either lifted or reinstated) after the study start date were censored at the time of their subsequent policy change (e.g., no longer contributed data).

#### Data analysis: target trial framework

Inverse probability weighting was used to adjust for potential time-varying confounding and selection bias due to loss to follow-up and censored at time when masking policy changed after study start. Inverse-probability treatment weighting (IPTW) addresses confounding by first using propensity scores to estimate the probability of receiving treatment given a set of baseline covariates and then each participant is assigned weights that is inversely proportional to their probability of receiving treatment. These weights create a pseudopopulation where the distribution of the various covariates is similar between the control group and treatment group. After these steps are completed, a pooled outcome regression model is fit using the weighted dataset to estimate the treatment effect. The IPTW facilitates use of the full dataset such that data points that cannot be matched are not discarded, as would occur when a matching method is applied. Additionally, IPTW also allows for adjusting of time-varying covariates.

The per-protocol analysis was conducted using inverse-probability weighting (IPW) models adjusted for U.S. region, urban/rural status, google mobility trends, county-level vaccination rate, county risk level, and in-school testing and vaccination programs. A *p*-value of <0.05 was used to determine statistical significance. To account for a possible delay in mask policy change impact, a sensitivity analysis with an 8-week follow up period was also estimated.

All analyses were completed using Python (Python Software Foundation) and STATA 17 (Statacorp). Annotated code underlying the cohort creation and analysis is included in Supplementary material and is also available on Github. A Strengthening the Reporting of Observational Studies in Epidemiology (STROBE) Checklist for Cohort studies is included in as a Supplementary material.

#### Ethical considerations

This study was approved as non-human subjects research by the VA Boston Research and Development committee prior to data collection and analysis.

## Results

### Event study

Out of 18,993 US Census Bureau-defined school districts in the US (25), 6,910 districts (36.4%) in 1,590 counties in 50 states had masking policy data available. Of these 6,910 districts, 3,970 underwent a policy change during the study period and were included. Included districts were composed of 53,453 schools and approximately 31,264,546 students.

Baseline demographics and county-level regional data about the included districts are presented in Table 2. Residents of included counties were 84.2% white and 9.4% black, and all four US census regions are represented. The most commonly utilized testing policy in school districts was none (70%), followed by partial (25.4%). The most common vaccination requirement in school districts was no mandate, followed by mandates for eligible populations.

**Table 2:** Demographics of the cohort (event study).

Variable		Included counties (%)	Included districts (N)
Population age^*^	0–9-year-old	N/A	18,472,242
	10–19-year-old	N/A	20,059,793
	20–49-year-old	N/A	24,881,822
	50–69-year-old	N/A	25,840,297
	≥70-year-old	N/A	25,683,790
Race^*^	White	78.36%	N/A
	American Indian	1.62%	N/A
	Black	12.56%	N/A
	Other	2.86%	N/A
	Asian^€^	4.6%	N/A
Poverty level^*^	Federal poverty level less than 100%	17.53%	N/A
Region^*^	Northeast	N/A	4,236,959
	Midwest	N/A	4,629,435
	South	N/A	7,157,255
	West	N/A	8,286,111
Vaccination uptake^*^^€^ (%)	5–11-year-old	25%	N/A
	12–17-year-old	56%	N/A
	18–64-year-old	67%	N/A
	≥65-year-old	87%	N/A
Other non-pharmaceutical interventions in schools	Student asymptomatic testing policy		
	Partial	N/A	7,561,980
	Selected populations	N/A	1,287,996
	Unvaccinated only	N/A	124,128
	None	N/A	20,798,505
	School vaccination policy		
	None	N/A	25,560,378
	Eligible Population	N/A	766,809
	High School Limited	N/A	521

* Variables presented at the county level.

€ Proportion of population reported as fully vaccinated as of the date of the masking policy de-adoption.

Unadjusted mean case rates per 100,000 county residents and hospitalization and death rates per 1,000,000 county residents stratified by decade of age are presented in Supplementary Figures 1–6. Prior to the policy change, there was not a clear trend in case rates in any of the age categories evaluated. Unadjusted mean rates in districts with schools that lifted masking requirements during October versus those that lifted during March are presented in Supplementary Figures 7–11. In these unadjusted analyses, districts with earlier lifting had consistently higher case, hospitalization, and death rates than schools that lifted masking requirements during the later parts of the school year. An association between early lifting of masking requirements in schools and higher levels of community recreational mobility was also identified (Supplementary Figure 12).

In the adjusted model, in school-mitigation measures significantly associated with community case rates included in-school vaccination requirements and asymptomatic testing policies. A higher proportion of individuals of Native American race was positively associated with community SARS-CoV-2 case rates and higher proportions of black and other non-White races were negatively associated with community-level SARS-CoV-2 cases. Higher poverty rates were positively associated with SARS-CoV-2 cases in the community. Higher levels of community retail and recreational mobility were positively associated with increases in cases and hospitalizations in all age groups studied.

In the adjusted event study analysis, sustained trends for increases in case rates were not identified for the whole cohort or for any age group (Table 3). For the whole cohort, there was a statistically significant increase in hospitalizations found 6–8 weeks following the policy change (maximum increase 1.91 hospitalizations per 1,000,000 county residents); increases in hospitalizations were also found in the stratified analysis for all age groups, although absolute impacts were small; magnitude of effect was highest among individuals >70 years of age (Table 4). Among individuals aged 0–9, the peak increase occurred 9 weeks following the policy change and was 1.02 hospitalizations per 1,000,000 county residents (95% CI, 0.73–1.32). For 10–19 year olds, the increases occurred from week 4 to week 10 following the policy change and point estimates ranged from 0.16 to 0.63. Trends for adults were similar but point estimates higher (for 20–49 year olds, 0.6–2.93; for 50–69 year olds, 0.83–4.1; for >70 year olds, 0.83–4.33).

**Table 3:** Event study: weekly change in SARS-CoV-2 cases per 100,000 following in-school masking policy changes.

Variable	Whole cohort	0–9 Year olds	10–19 Year olds	20–49 Year olds	50–69 Year olds	70+ Year olds
Fully vaccinated proportion	**−3.86 [−5.15, −2.58]**	**−0.00004 [−0.00004, −0.00003]**	**−0.00002 [−0.00002, −0.00002]**	**−0.000003 [−0.000003, −0.000002]**	**−0.000002 [−0.000002, −0.000002]**	**−6.5 [−9.08, −3.93]**
Month	**−0.2 [−0.22, −0.18]**	**−0.16 [−0.18, −0.14]**	**−0.09 [−0.11, −0.07]**	**−0.21 [−0.27, −0.15]**	**−0.12 [−0.16, −0.08]**	**−0.2 [−0.23, −0.17]**
Student asymptomatic testing policy^a^						
Unvaccinated only	**−3.4 [−4.25, −2.56]**			**2.84 [1.3, 4.38]**	**2.43 [1.44, 3.41]**	**−2.34 [−3.87, −0.8]**
Partial or optional	**−0.69 [−0.98, −0.39]**	0.2 [−0.09, 0.5]	−0.13 [−0.44, 0.19]	**−1.01 [−1.79, −0.23]**	**−0.71 [−1.2, −0.22]**	**−0.96 [−1.28, −0.63]**
Selected populations only	**−1.25 [−2, −0.49]**	0.22 [−0.39, 0.83]	−0.24 [−1.09, 0.61]	−0.96 [−3.36, 1.43]	−0.48 [−1.75, 0.79]	**−1.23 [−2.17, −0.3]**
Student vaccination requirement^b^						
High school	**−5.07 [−5.39, −4.75]**			**1.35 [0.18, 2.52]**	**1.32 [0.58, 2.07]**	**−4.4 [−4.74, −4.06]**
Eligible populations	**−1.81 [−2.45, −1.17]**	**0.31 [0.01, 0.6]**	−0.21 [−0.57, 0.15]	1.57 [−0.63, 3.78]	0.88 [−0.61, 2.36]	−0.71 [−1.61, 0.19]
Racial composition of county^c^						
Black Race	**−0.04 [−0.05, −0.03]**	**−0.02 [−0.03, −0.01]**	**−0.02 [−0.03, −0.01]**	**−0.05 [−0.08, −0.02]**	**−0.05 [−0.06, −0.03]**	**−0.05 [−0.07, −0.04]**
Other Race	**−0.87 [−1.02, −0.72]**	**−0.58 [−0.77, −0.39]**	**−0.61 [−0.86, −0.36]**	**−1.14 [−1.59, −0.69]**	**−1.01 [−1.28, −0.73]**	**−0.76 [−0.9, −0.63]**
Native American	**0.08 [0.05, 0.11]**	**0.02 [0.01, 0.04]**	**0.04 [0.01, 0.07]**	**0.08 [0.01, 0.16]**	**0.07 [0.03, 0.12]**	**0.03 [0, 0.05]**
Poverty level below 100%	**−0.11 [−0.14, −0.09]**	**−0.12 [−0.15, −0.08]**	**−0.11 [−0.15, −0.07]**	**−0.28 [−0.37, −0.19]**	**−0.17 [−0.23, −0.12]**	−0.02 [−0.06, 0.01]
Mobility movement						
Grocery	**0.13 [0.12, 0.14]**	**0.07 [0.06, 0.08]**	**0.06 [0.05, 0.07]**	**0.22 [0.19, 0.26]**	**0.12 [0.1, 0.14]**	**0.09 [0.07, 0.1]**
Park	**−0.003 [−0.01, −0.001]**	**−0.004 [−0.006, −0.003]**	**−0.01 [−0.01, 0]**	**−0.02 [−0.03, −0.02]**	**−0.01 [−0.02, −0.01]**	−0.002 [−0.004, 0]
Transit	**0.03 [0.02, 0.04]**	**0.004 [−0.001, 0.008]**	**0.02 [0.01, 0.03]**	**0.05 [0.03, 0.07]**	**0.03 [0.02, 0.05]**	**0.03 [0.02, 0.03]**
Workplace	**0.01 [0.01, 0.02]**	**0.01 [0.01, 0.02]**	**0.01 [0, 0.02]**	**0.02 [0, 0.04]**	0.01 [0, 0.02]	0.002 [−0.01, 0.01]
−4	**1.82 [1.5, 2.14]**	**0.94 [0.56, 1.31]**	**0.99 [0.6, 1.38]**	**2.35 [1.33, 3.37]**	**1.79 [1.24, 2.34]**	**2.07 [1.68, 2.45]**
−3	**0.61 [0.27, 0.95]**	**0.63 [0.26, 1]**	**0.52 [0.09, 0.96]**	0.94 [−0.18, 2.05]	**0.83 [0.16, 1.51]**	**0.99 [0.51, 1.47]**
−2	**0.74 [0.46, 1.02]**	**0.47 [0.16, 0.77]**	**0.44 [0.07, 0.81]**	**0.96 [0.06, 1.85]**	**0.82 [0.28, 1.36]**	**0.82 [0.43, 1.22]**
−1	**0.6 [0.4, 0.8]**	**0.35 [0.1, 0.6]**	**0.33 [0.11, 0.56]**	**0.9 [0.28, 1.52]**	**0.77 [0.36, 1.18]**	**0.63 [0.32, 0.95]**
1	**−0.25 [−0.46, −0.04]**	−0.07 [−0.33, 0.2]	0.24 [−0.05, 0.53]	0.08 [−0.59, 0.75]	0.06 [−0.33, 0.45]	−0.2 [−0.48, 0.07]
2	**−0.31 [−0.53, −0.08]**	−0.13 [−0.42, 0.17]	0.16 [−0.14, 0.47]	0.07 [−0.65, 0.79]	0.07 [−0.34, 0.48]	−0.2 [−0.49, 0.09]
3	−0.1 [−0.34, 0.14]	0.05 [−0.26, 0.36]	**0.43 [0.09, 0.76]**	0.4 [−0.37, 1.18]	0.3 [−0.15, 0.74]	−0.12 [−0.42, 0.19]
4	−0.14 [−0.39, 0.12]	0.06 [−0.24, 0.37]	0.18 [−0.15, 0.51]	0.25 [−0.57, 1.08]	0.46 [−0.03, 0.94]	0.05 [−0.26, 0.37]
5	0.27 [−0.01, 0.55]	0.37 [0.03, 0.71]	**0.53 [0.19, 0.88]**	0.87 [−0.06, 1.8]	**1.05 [0.51, 1.59]**	0.25 [−0.08, 0.58]
6	−0.07 [−0.33, 0.2]	0.12 [−0.21, 0.45]	0.17 [−0.18, 0.52]	0.06 [−0.81, 0.92]	**0.67 [0.17, 1.17]**	0.14 [−0.19, 0.46]
7	**0.48 [0.21, 0.75]**	**0.51 [0.17, 0.85]**	**0.55 [0.19, 0.91]**	**0.97 [0.08, 1.86]**	**1.31 [0.79, 1.83]**	**0.62 [0.28, 0.96]**
8	**−0.68 [−0.96, −0.4]**	−0.03 [−0.37, 0.31]	−0.01 [−0.38, 0.37]	**−1.16 [−2.06, −0.27]**	−0.06 [−0.58, 0.45]	−0.12 [−0.45, 0.22]

Variables in bold, *p* < 0.05. All output represents change in SARS-CoV-2 cases per 100,000 county residents. Estimates in brackets represent the 95% confidence intervals.

**Table 4:** Event study: SARS-CoV-2 hospitalizations per 1,000,000 residents.

Variable	Whole cohort	0–9 Year olds	10–19 Year olds	20–49 Year olds	50–69 Year olds	70+ Year olds
Fully vaccinated proportion	**−13.51 [−15.71, −11.31]**	**−0.00001 [−0.00001, −0.00001]**	**−0.000004 [−0.000005, −0.000003]**	**−0.000003 [−0.000003, −0.000003]**	**−0.000004 [−0.000005, −0.000004]**	**−18.94 [−28.29, −9.59]**
Month	**−0.3 [−0.35, −0.26]**	**−0.16 [−0.18, −0.14]**	**−0.09 [−0.1, −0.07]**	−0.06 [−0.14, 0.02]	**0.13 [0.02, 0.24]**	**−0.49 [−0.61, −0.38]**
Student asymptomatic testing policy^a^						
Unvaccinated only	**−7.77 [−9.35, −6.19]**			−0.74 [−2.44, 0.96]	1.84 [−0.7, 4.38]	**−11.8 [−17.44, −6.16]**
Partial or optional	**−1.55 [−2.05, −1.04]**	0.05 [−0.32, 0.42]	−0.23 [−0.47, 0.01]	**−2.89 [−3.79, −2]**	**−3.41 [−4.29, −2.53]**	**−4.98 [−6.01, −3.94]**
Selected populations only	**−2.98 [−4.52, −1.44]**	−0.39 [−1.72, 0.94]	**−0.86 [−1.34, −0.38]**	−2.74 [−5.61, 0.13]	−2.86 [−5.78, 0.06]	**−5.74 [−9.14, −2.33]**
Student vaccination requirement^b^						
High school	**−10.94 [−11.93, −9.95]**			**−2.67 [−3.92, −1.42]**	−0.54 [−2.16, 1.09]	**−19.64 [−21.15, −18.13]**
Eligible populations	**−3.81 [−5.42, −2.19]**	−0.27 [−0.59, 0.05]	0.02 [−0.3, 0.34]	−0.2 [−1.28, 0.89]	0.4 [−1.87, 2.68]	**−5.29 [−8.66, −1.92]**
Racial composition of county^c^						
Black Race	**−0.06 [−0.08, −0.04]**	**0.02 [0.01, 0.03]**	**0.02 [0.02, 0.03]**	**0.04 [0, 0.07]**	**−0.1 [−0.13, −0.06]**	**−0.18 [−0.22, −0.13]**
Other Race	**−0.93 [−1.22, −0.63]**	**−0.16 [−0.31, −0.01]**	−0.05 [−0.16, 0.07]	−0.54 [−1.12, 0.04]	**−1.41 [−2.07, −0.76]**	**−1.9 [−2.51, −1.29]**
Native American	**0.06 [0.02, 0.1]**	**0.04 [0.03, 0.06]**	−0.01 [−0.01, 0]	−0.03 [−0.08, 0.03]	0.02 [−0.04, 0.09]	−0.02 [−0.08, 0.05]
Poverty level below 100%	**−0.43 [−0.49, −0.37]**	**−0.16 [−0.19, −0.13]**	**−0.13 [−0.16, −0.11]**	**−0.71 [−0.85, −0.57]**	**−0.67 [−0.8, −0.54]**	**−0.24 [−0.36, −0.11]**
Mobility movement						
Grocery	**0.11 [0.09, 0.13]**	**0.03 [0.02, 0.03]**	**0.02 [0.01, 0.02]**	**0.09 [0.05, 0.13]**	**0.09 [0.05, 0.14]**	**0.18 [0.13, 0.22]**
Park	−0.001 [−0.004, 0.002]	−0.0003 [−0.002, 0.001]	0.001 [−0.001, 0.002]	**−0.02 [−0.02, −0.01]**	**−0.04 [−0.05, −0.03]**	**−0.01 [−0.02, 0]**
Transit	**0.01 [0.01, 0.02]**	**−0.01 [−0.01, −0.004]**	**−0.004 [−0.01, −0.0005]**	0.01 [−0.01, 0.02]	**0.05 [0.02, 0.07]**	**0.07 [0.04, 0.1]**
Workplace	−0.01 [−0.02, 0]	0.001 [−0.002, 0.005]	0.0002 [−0.003, 0.004]	0.002 [−0.02, 0.02]	−0.01 [−0.04, 0.01]	−0.01 [−0.03, 0.02]
−4	**3.89 [3.2, 4.58]**	**0.44 [0.21, 0.67]**	**0.42 [0.24, 0.6]**	**3.86 [2.74, 4.99]**	**6.01 [4.71, 7.3]**	**10.33 [9.01, 11.65]**
−3	**1.42 [0.68, 2.16]**	0.1 [−0.26, 0.46]	0.36 [−0.02, 0.75]	0.59 [−0.79, 1.97]	1.07 [−0.39, 2.52]	**4.71 [3.06, 6.36]**
−2	**1.06 [0.44, 1.69]**	0.16 [−0.24, 0.56]	0.21 [−0.12, 0.54]	0.35 [−0.75, 1.46]	0.91 [−0.35, 2.18]	**2.91 [1.57, 4.25]**
−1	**0.65 [0.14, 1.15]**	**−0.36 [−0.63, −0.08]**	−0.1 [−0.32, 0.12]	−0.2 [−1.24, 0.83]	0.19 [−0.95, 1.34]	**1.63 [0.27, 2.99]**
1	**−0.65 [−1.14, −0.16]**	−0.13 [−0.32, 0.06]	0.12 [−0.03, 0.28]	0.43 [−0.08, 0.95]	0.29 [−0.28, 0.85]	0.35 [−0.34, 1.03]
2	−0.42 [−0.94, 0.11]	−0.05 [−0.27, 0.18]	**0.16 [0, 0.32]**	**0.6 [0.02, 1.18]**	**0.83 [0.16, 1.51]**	**0.83 [0.13, 1.54]**
3	−0.18 [−0.74, 0.38]	0.2 [−0.02, 0.42]	0.06 [−0.11, 0.23]	**0.76 [0.09, 1.43]**	**1.7 [0.83, 2.57]**	**1.59 [0.79, 2.4]**
4	0.28 [−0.31, 0.86]	0.19 [−0.04, 0.43]	**0.19 [0.02, 0.36]**	**0.81 [0.13, 1.49]**	**1.98 [1.1, 2.86]**	**1.72 [0.87, 2.57]**
5	0.43 [−0.16, 1.03]	**0.36 [0.11, 0.62]**	**0.28 [0.11, 0.45]**	**0.98 [0.33, 1.63]**	**1.92 [1.15, 2.68]**	**1.62 [0.85, 2.4]**
6	**0.66 [0.09, 1.24]**	**1.01 [0.72, 1.29]**	**0.43 [0.22, 0.65]**	**2.24 [1.48, 3]**	**3.48 [2.58, 4.38]**	**3.87 [2.96, 4.78]**
7	**1.91 [1.3, 2.52]**	**1.02 [0.73, 1.32]**	**0.63 [0.4, 0.85]**	**2.93 [1.94, 3.92]**	**4.01 [2.75, 5.27]**	**4.33 [3.08, 5.59]**
8	0.06 [−0.52, 0.63]	**0.4 [0.18, 0.61]**	**0.26 [0.09, 0.43]**	−0.15 [−0.79, 0.49]	0.63 [−0.12, 1.39]	1.35 [0.56, 2.13]

Variables in bold, *p* < 0.05. All output represents change in SARS-CoV-2 hospitalizations per 1,000,000 county residents. Estimates in brackets represent the 95% confidence intervals.

Adjusted impacts on COVID-19 deaths are presented in Table 5. An increase in deaths was found during the period from 4 to 7 weeks following the policy change (maximum increase, 0.62 deaths per 1,000,000 residents). No increases in deaths were identified for individuals ≤19 years of age and there were no clear trends among individuals <50. Among 50–69 year olds, a small but inconsistent increase was found with point estimates ranging from 0.088 to 1.49. For >70 year olds, increases were found for most weeks, ranging from 0.23 to 2.58 cases.

**Table 5:** Event study: COVID-19 deaths per 1,000,000 county residents following in-school masking policy changes.

Variable	Whole cohort	0–9 Year olds	10–19 Year olds	20–49 Year olds	50–69 Year olds	70+ Year olds
Fully vaccinated proportion	**−9.15 [−10.34, −7.96]**	**−0.0000005 [−0.0000007, −0.0000003]**	**0.0000005 [0.0000003, 0.0000006]**	**−0.0000002 [−0.0000002, −0.0000002]**	**−0.0000011 [−0.0000013, −0.000001]**	**−22.02 [−27.79, −16.24]**
Month	**−0.05 [−0.08, −0.03]**	**−0.003 [−0.005, −0.001]**	**−0.009 [−0.013, −0.006]**	**0.058 [0.037, 0.079]**	**0.228 [0.176, 0.28]**	−0.06 [−0.13, 0.01]
Student asymptomatic testing policy^a^						
Unvaccinated Only	**−1.15 [−2.08, −0.21]**					**−4.73 [−8.32, −1.15]**
Partial or Optional	**−0.17 [−0.34, 0]**	**−0.01 [−0.02, −0.003]**	**−0.03 [−0.04, −0.021]**	**−0.63 [−0.72, −0.544]**	**−1.78 [−2.07, −1.491]**	**−2.4 [−2.81, −1.99]**
Selected Populations Only	−0.24 [−0.82, 0.35]	0.0003 [−0.03, 0.03]	0.01 [−0.09, 0.11]	**−0.45 [−0.69, −0.2]**	**−0.9 [−1.71, −0.09]**	**−1.84 [−3.47, −0.21]**
Student vaccination requirement^b^						
High School	**−2.44 [−3.35, −1.53]**					**−9.63 [−10.32, −8.95]**
Eligible populations	0.2 [−0.48, 0.88]	**0.01 [0.003, 0.03]**	**0.04 [0.019, 0.06]**	**−0.3 [−0.507, −0.09]**	0.13 [−0.461, 0.72]	−0.93 [−2.95, 1.1]
Racial composition of county^c^						
Black Race	**−0.06 [−0.07, −0.05]**	−0.0004 [−0.001, 0.0003]	**0.0014 [0.001, 0.0022]**	**−0.0072 [−0.012, −0.0021]**	**−0.0611 [−0.075, −0.0471]**	**−0.18 [−0.21, −0.15]**
Other Race	**−0.61 [−0.74, −0.48]**	**−0.02 [−0.04, −0.003]**	−0.01 [−0.03, 0.009]	**−0.34 [−0.44, −0.231]**	**−0.96 [−1.19, −0.737]**	**−1.25 [−1.53, −0.98]**
Native American	**0.1 [0.07, 0.14]**	−0.001 [−0.002, 0.00004]	**−0.002 [−0.002, −0.00087]**	**0.035 [0.027, 0.0437]**	**0.135 [0.082, 0.18905]**	**0.13 [0.07, 0.18]**
Poverty level below 100%	**−0.07 [−0.09, −0.05]**	−0.002 [−0.003, 0.00003]	**−0.004 [−0.006, −0.00194]**	**−0.038 [−0.052, −0.02367]**	**−0.076 [−0.112, −0.03909]**	0.04 [−0.01, 0.09]
Mobility movement						
Grocery	**0.01 [0, 0.02]**	−0.001 [−0.003, 0.00041]	**0.002 [0.001, 0.00291]**	**0.011 [0.004, 0.01788]**	−0.009 [−0.027, 0.00832]	0.01 [−0.01, 0.04]
Park	**−0.01 [−0.01, −0.01]**	**−0.0002 [−0.0003, −0.0001]**	**−0.0004 [−0.0006, −0.0003]**	**−0.0109 [−0.0119, −0.00982]**	**−0.0347 [−0.0376, −0.03173]**	**−0.04 [−0.04, −0.03]**
Transit	**0.01 [0.01, 0.02]**	0.0005 [−0.0003, 0.001]	**0.0013 [0.0008, 0.002]**	**0.0114 [0.0081, 0.015]**	**0.0374 [0.0259, 0.049]**	**0.05 [0.04, 0.07]**
Workplace	**−0.01 [−0.02, −0.01]**	−0.0004 [−0.001, 0.00023]	**−0.0011 [−0.002, −0.00055]**	**−0.0072 [−0.011, −0.00311]**	**−0.0239 [−0.035, −0.0129]**	−0.01 [−0.03, 0]
−4	**1.23 [0.92, 1.54]**	−0.005 [−0.03, 0.02]	**0.026 [0, 0.05]**	**0.565 [0.36, 0.77]**	**1.778 [1.32, 2.24]**	**5.75 [5.08, 6.43]**
−3	**0.44 [0.09, 0.78]**	−0.02 [−0.06, 0.02]	0.01 [−0.06, 0.08]	−0.14 [−0.4, 0.12]	−0.001 [−0.64, 0.64]	**2.25 [1.34, 3.16]**
−2	0.22 [−0.05, 0.48]	**−0.04 [−0.07, −0.02]**	0.04 [−0.02, 0.09]	0.12 [−0.16, 0.4]	0.45 [−0.23, 1.13]	**1.58 [0.73, 2.44]**
−1	**0.32 [0.08, 0.56]**	−0.03 [−0.06, 0.003]	−0.02 [−0.05, 0.004]	**−0.26 [−0.44, −0.075]**	−0.17 [−0.67, 0.321]	**0.91 [0.13, 1.69]**
1	−0.17 [−0.4, 0.07]	−0.02 [−0.04, 0.002]	0.01 [−0.02, 0.041]	−0.04 [−0.22, 0.139]	−0.02 [−0.3, 0.261]	0.23 [−0.23, 0.69]
2	−0.1 [−0.34, 0.14]	−0.004 [−0.03, 0.03]	−0.018 [−0.04, 0]	0.182 [0, 0.37]	**0.502 [0.13, 0.87]**	**0.58 [0.1, 1.06]**
3	0.09 [−0.17, 0.35]	**−0.03 [−0.05, −0.01]**	0.01 [−0.02, 0.03]	**0.34 [0.1, 0.57]**	**0.68 [0.27, 1.09]**	**0.84 [0.37, 1.31]**
4	**0.29 [0.03, 0.55]**	0.01 [−0.08, 0.09]	−0.005 [−0.04, 0.03]	**0.212 [0.02, 0.4]**	**1.267 [0.72, 1.81]**	**1.16 [0.56, 1.76]**
5	**0.48 [0.19, 0.76]**	**−0.03 [−0.05, −0.004]**	−0.012 [−0.03, 0.01]	0.127 [−0.04, 0.3]	0.164 [−0.23, 0.55]	**0.72 [0.23, 1.2]**
6	0.2 [−0.05, 0.46]	**−0.02 [−0.04, −0.0004]**	**−0.02 [−0.04, 0]**	−0.019 [−0.23, 0.19]	**0.798 [0.37, 1.23]**	**2.04 [1.42, 2.66]**
7	**0.62 [0.34, 0.9]**	−0.01 [−0.04, 0.02]	−0.001 [−0.03, 0.02]	0.276 [−0.03, 0.58]	**1.489 [0.74, 2.23]**	**2.58 [1.38, 3.77]**
8	0.08 [−0.17, 0.34]	−0.02 [−0.04, 0.002]	0.001 [−0.02, 0.02]	−0.11 [−0.28, 0.06]	0.088 [−0.27, 0.45]	1.14 [0.65, 1.63]

Variables in bold, *p* < 0.05. All output represents change in SARS-CoV-2 deaths per 1,000,000 county residents. Estimates in brackets represent the 95% confidence intervals.

In the sensitivity analyses with 0 and 2-week lags, overall impacts on cases were similar. For the 0 week lag, differences between districts that lifted versus maintained their mask mandates were generally less than 1 case per 100,000 county residents per week. For the 2-week lag, the effect estimates were of larger magnitude than for the model estimated with a 1-week lag but largely remained insignificant. More details are presented in Supplementary Tables 2–4.

### Observational cohort with a target trial emulation framework

**Figure 3:**
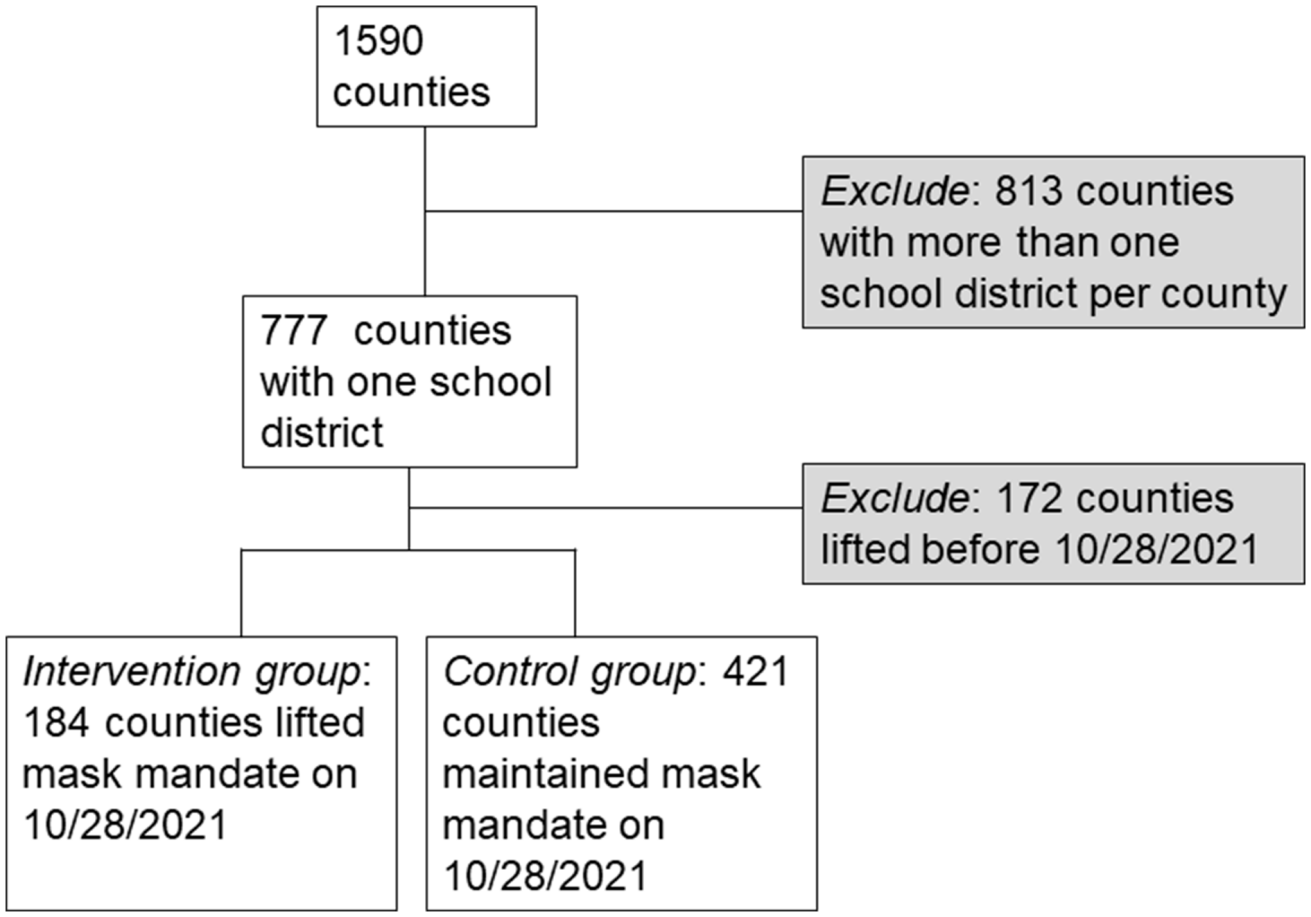
Cohort flow diagram for included and excluded counties. Description of cohort inclusion and exclusion criteria.

Out of 1,590 US counties with complete data available, 777 had only one school district (Supplementary Figures 13, 14). Of these, *N* = 172 did not have a masking requirement on 10/28/2021 and were ineligible for inclusion. Of the remaining 605 districts, 184 intervention districts with a total school population of 837,075 lifted their masking requirement on 10/28/2021. 421 control districts with a total school population of 6,572,087 that maintained their masking requirement (See Figure 3 for cohort flow diagram, Supplementary Figure 15 for mask mandate removal dates among included counties). Due to universal masking requirements in the region during the study period, no counties in the Northeast were represented in the intervention group (see Supplementary Table 5 for demographic details of excluded counties).

Demographics of intervention and control counties are presented in Table 6. Overall, intervention counties had slightly higher proportions of White residents (89.9% versus 82.9%) and slightly lower Black residents (4.4% versus 9.5%). Vaccination uptake was higher in control versus intervention counties for every age group. All categories of community mobility were higher in in intervention counties versus controls.

**Table 6:** Demographics of intervention and control counties included in the target trial emulation framework analysis.

Variable		Intervention counties	Control counties
		Included counties (%)/included districts (school population)	Included counties (%)/included districts (school population)
Total school population		832,780	6,572,087
Race^*^	White	89.89%	82.86%
	American Indian	2.39%	3.07%
	Black	4.4%	9.54%
	Other	2.23%	2.41%
	Asian^€^	1.09%	2.11%
Poverty level^*^	Federal poverty level less than 100%	21%	20.82%
Region^*^	Northeast	0	63,282
	Midwest	102,702	340,305
	South	696,051	5,265,267
	West	34,027	903,233
Urban/Rural	Urban	476,951	5,629,636
	Suburban	240,685	604,050
	Rural	115,144	338,401
Mobility	Grocery	4.79%	3.03%
	Park	12.19%	7.4%
	Residential	5.6%	3.83%
	Retail	1.14%	−2.04%
	Transit	7.7%	−2.66%
	Workplace	−20.66%	−19.68%
Vaccination uptake^*^^€^ (%)	5–11-year-old	8.56%	16.09%
	12–17-year-old	28.17%	42.6%
	18–64-year-old	48.52%	57.57%
	≥65-year-old	76.76%	81.42%
Other non-pharmaceutical interventions in schools	Student asymptomatic testing policy		
	Partial	160,589	1,658,076
	Selected populations	0	1,089,398
	Unvaccinated only	0	77,807
	None	832,780	5,951,267
	School vaccination policy		
	None	832,780	6,572,087
	Eligible Population	0	0
	High School Limited	0	0

* Variables presented at the county level.

€ Proportion of population reported as fully vaccinated as of the date of the masking policy de-adoption.

Over the course of the 8-week study period, 43 counties switched their policy and were censored at the time of the policy change (38 control counties lifted their masking mandate and 5 counties in the intervention group re-instated their masking policy). The distribution of cases, hospitalizations, and deaths stratified by age group in intervention and control counties during the study period is presented in Supplementary Table 6. The total number of deaths in individuals aged 0–9 was 6 in the intervention counties and 18 in the control counties. Among individuals aged 10–19, there were 20 deaths in intervention counties and 24 deaths in the control counties. Aligned with the well-recognized age distribution of COVID-19 severity, death rates in older individuals (>70) were substantially higher in both intervention (*N* = 696 deaths) and control (*N* = 1,646 deaths) when compared to the school-aged population.

In the unadjusted analysis, cases, hospitalizations and deaths were increased in the counties that lifted masking mandates versus those that did not lift masking mandates (Supplementary Table 7); increases in hospitalizations and deaths for the whole cohort were driven by severe outcomes in individuals 50 years of age and older.

Overall incidence of hospitalization and deaths were higher in older individuals relative to the younger cohort. Across the whole cohort, there were no increases in cases, hospitalizations, or deaths following the policy change. In the regression models, adjusted for county-level vaccination rates, county SARS-CoV-2 risk level, and community mobility, the mean difference in case rates per 100,000 county residents over the 8-week period in intervention and control counties was −1.06 (95% CI, −1.85, −0.27) (Table 7). Hospitalization rates (−1.50, 95% CI, −3.97, 0.72) and death rates (−0.94, 95% CI, −2.27, 0.38) per 1,000,000 county residents were also similar. Findings were similar when stratified by age. The one exception was a small increase in deaths (<1/1,000,000 county residents) among 10–19 year olds. Even among this group, increases were not demonstrated for cases or hospitalizations.

**Table 7:** Target trial emulation framework: average impact of lifting of mask mandates in US counties with one school district per county during the 8-weeks following the policy change.

Outcome	Strata	Mask mandate lifted (intervention)	Mask mandate maintained (control)	Difference^*^	95% Confidence Interval		*p*-value
Cases per 100,000 county residents	Whole cohort	4.57	5.63	**−1.06**	**−1.85**	**−0.27**	**0.01**
	0–9 year olds	2.38	3.10	**−0.71**	**−1.30**	**−0.13**	**0.02**
	10–19 year olds	2.65	3.36	−0.71	−1.60	0.17	0.11
	20–49 year olds	8.11	8.86	−0.75	−2.91	1.41	0.49
	50–69 year olds	5.35	6.31	−0.96	−2.92	0.99	0.33
	70+ year olds	3.52	5.17	**−1.65**	**−2.96**	**−0.34**	**0.01**
Hospitalizations per 1,000,000 county residents	Whole Cohort	8.88	10.38	−1.50	−3.72	0.72	0.19
	0–9 year olds	1.01	0.92	0.09	−0.55	0.73	0.78
	10–19 year olds	0.89	1.59	−0.70	−1.94	0.54	0.27
	20–49 year olds	8.47	9.09	−0.62	−4.03	2.79	0.72
	50–69 year olds	14.94	15.21	−0.27	−6.16	5.62	0.93
	70+ year olds	16.89	23.42	−6.54	−15.43	2.35	0.15
Deaths per 1,000,000 county residents	Whole Cohort	4.04	4.98	−0.94	−2.27	0.38	0.16
	0–9 year olds	0.05	0.31	−0.27	−0.81	0.27	0.33
	10–19 year olds	0.36	0.05	**0.30**	**0.04**	**0.57**	**0.03**
	20–49 year olds	2.51	3.27	−0.76	−2.54	1.02	0.40
	50–69 year olds	6.42	7.08	−0.66	−3.97	2.65	0.70
	70+ year olds	10.02	14.61	−4.59	−12.22	3.04	0.24

Variables in bold, *p* < 0.05.

* A negative difference indicates a fall following the policy change; i.e., lower cases, hospitalizations, and deaths following lifting of in-school mask mandates. Positive differences indicate increases following the policy change.

Results were similar but generally with larger effect estimates in the sensitivity analysis that extended the follow up period to 8 weeks (Supplementary Table 8). Major differences included a statistically significant decrease in cases among counties that lifted their mask mandate (*p* = 0.04) and a statistically significant increase in deaths in 10–19 year olds (difference, 0.11/1,000,000 count residents, *p* = 0.05).

## Discussion

Schools began re-opening across the United States starting in the summer of 2020 with variable SARS-CoV-2 mitigation measures including physical distancing, curtailing of extracurricular activities, use of plexiglass, and, in many cases, maintenance of fully or partially remote learning. Subsequently, vaccines became widely available to all adults and then for primary and secondary school aged students, testing access and capacity expanded, and population-level immunity increased, particularly following the emergence of the Omicron variant. As these changes occurred, the CDC updated its in-school mitigation guidelines, and policies about non-pharmaceutical interventions were variably lessened across the country. In many areas, in-school masking requirements were among the last policies to be retired, yet the downstream impacts of this policy change on the burden of COVID-19 disease remained unknown. Lifting of masking policies in schools was particularly controversial, given prior modeling studies predicting that in-school transmission would be a major catalyst for community transmission and theoretical concerns that lifting masking requirements in schools would have a spillover effect on the surrounding community (4), leading to increases in cases in older adults and persons with immunocompromising conditions, who remain at higher risk of severe disease despite vaccination (26). In this nationwide retrospective cohort event study analysis conducted during the period after medical countermeasures were widely available for adults, we found no impact on SARS-CoV-2 cases, very small increases in hospitalizations across all age groups, and slight increases in deaths among individuals 50 years of age and older, with impacts highest in those >70, consistent with the well-described age-dependent risk of severe outcomes following SARS-CoV-2 infection and overall risk of death. When a TTE framework was applied, minimal differences were seen among those who had masking policy lifted and stayed unmasked and those who continued masking mandate through the study period.

This retrospective, observational study used two different analytic designs: an event study framework and a TTE framework. Both approaches have advantages and disadvantages. The event study framework includes a more diverse representation of school districts and counties, including urban school districts and schools in the Northeast in the intervention group, which tended to have more restrictive mitigation policies than schools in other regions. The event study framework also spanned a longer period of time, thus including more variation in terms of circulating variants and community. However, the limitation of this approach is that districts within counties may have adopted different policies, the method might not appropriately account for temporal trends, and despite the difference in difference model, uncontrolled confounding remains a concern. The TTE framework is designed to address several of these limitations: only counties with one district per county were included, there were strict inclusion and exclusion criteria, and the impact of the policy change was evaluated based on one date, aligning temporal changes with censoring at the time of changing treatment strategies, in this case at the time of deviating from the assigned masking policy at the time of study entry. However, the limitation of this approach is a smaller number of included counties and the lack of representation of counties from the Northeast in the intervention group. Given that there are limitations to both analytic approaches, we opted to present both. Notably, the different analyses lead to slightly different conclusions: in the event study, there appeared to be a small in magnitude but measurable community impact of lifting of mask mandates in schools but these trends were not found in the TTE framework, which did not find any statistically increase in any COVID-19 outcome in the whole cohort. This is aligned with other work highlighting the challenges of measuring the impacts of in-school mitigation measures (27). However, although the results are not perfectly congruent, both point to either no impact or a very small impact (on the order of 1/1,000,000) of lifting masks in schools on severe outcomes in the surrounding community. Additionally, in theory, in order to cause additional hospitalizations and deaths, COVID-19 case rates would have to increase and we did not identify an impact on case rates in either analysis. This raises the question of whether the hospitalizations and deaths are truly due to COVID or due to another cause.

The primary outcome of our study was county-level case rates, with hospitalization and death rates and age-stratified results evaluated as secondary outcomes. Early in the pandemic, case rates were highly correlated with hospitalization rates and SARS-CoV-2-related mortality; therefore, policy goals focused on delaying infection until medical countermeasures could be deployed. During the period following vaccine availability, there was a de-coupling of case rates from hospitalization rates and deaths (28–30). Since the COVID-19 emergency was officially declared over in May 2023, the primary aim of public health interventions has been to prevent severe disease and deaths, rather than focusing on non-pharmaceutical interventions that delay but do not ultimately prevent cases. It is important to consider the findings of this analysis in the context of current goals.

This study was conducted after a full academic year (2020–2021) of masking requirements in much of the country had elapsed, and after masking requirements had been lifted in most non-school settings. Both of these contextual factors may have impacted study findings. First, it is conceivable that the duration of the impact of masking policies is short-lived, due to adherence fatigue, changes in susceptibility to the pathogen, or other factors; prior work suggests that masking policy impacts are both condition and context dependent (8), and these are important factors when considering generalizability of findings beyond the specific timeframe included in the study. Second, it is possible that application of masking policies in limited settings such as schools but not more widely has minimal effect on case rates. Conversely, the effect estimate may have been significant if masking requirements were lifted in schools before other segments of society.

The association between lower community mobility and lower incidence of SARS-CoV-2 is not surprising. The lower incidence of cases in counties with more Black residents is somewhat unexpected, as this group generally fared worse than the overall population during the early phases of the pandemic and may be due to greater community immunity at the time of the study, due to a higher rate of infection earlier. The lower incidence of cases in counties with school-based testing programs suggests these may have played a beneficial role in protecting the surrounding community, though confounders, such as district resources, are numerous. Other studies with substantially more granular detail about in-school testing programs have not found a significant impact of surveillance testing on cases in students but did find that testing can be used as a strategy to support in-person learning (38).

Any potential benefits of masking requirements in schools need to be weighed against their potential downsides. Face masks interfere with verbal and non-verbal communication (31–33), which in turn, likely interferes with the effectiveness of in-person education. Additionally, face masks interrupt human connections, increase hearing efforts (31, 33), and have likely impacts on morale. It should be noted that SARS-CoV-2 related hospitalizations in this study represent both those that are attributable to COVID-19 and those that occur for unrelated reasons in patients who have asymptomatic or mild SARS-CoV-2. The proportion of hospitalizations for severe disease has decreased over time, as has the proportion of COVID-19 attributable deaths (28, 34–36). Thus, increases in these two metrics, particularly in the later periods of the study should be interpreted with caution; the attributable cases are likely even lower.

Our study has several limitations. First, this is a retrospective dataset subject to all of the limitations of non-randomized, observational data. It is possible our results are impacted by residual and unmeasured confounding or other factors not accounted for in our analysis. The TTE framework was designed to control for a variety of different factors, including calendar time and used an IPTW statistical technique. Although IPTW is a robust strategy for adjusting for confounding, it only takes into consideration the available variables for weighting and thus is still vulnerable to residual confounding from unmeasured variables. Neither event study designs nor TTE frameworks are able to completely eliminate biases that arise from a lack of randomization. We conducted multiple sensitivity analyses to test our methodologic decisions and assumptions and findings were robust to these changes, however, as noted above, results differed in small ways from our initial analyses. While a randomized controlled trial is a more robust causal design than either the event study or the TTE framework approach applied in this analysis, conducting a randomized controlled trial of in-school masking policies faces major feasibility challenges due to the nature of the school setting. Specifically, there are questions about consent for participation (which would require approval and buy-in from many stakeholders, including parents, teachers, and local school leadership), concerns about clinical equipoise and perceptions about the ethnical nature of participating in such a trial from stakeholders, questions about policy control and decision making, as well as practical considerations about assessment and tracking of outcomes, including standing up testing programs and measuring outcomes that occur outside of the school setting (such as hospitalization). Additionally, solving all of these challenges would only address the issue of masking in in-school settings. This study sought to evaluate the impact of implementing masking requirements in school settings on outcomes in the surrounding community. Even if masking policy in schools could be dictated as part of a randomized controlled trial, and outcomes among those participants attending school accurately tracked, systematic collection of outcomes data for community members not attending school would not be included in such an analysis (it would also require consent and outcomes reporting) and thus it is highly likely these questions would also be addressed using an observational study design.

A second limitation is that this study was conducted during a specific period during the pandemic when vaccines were widely available to all adults and secondary school-aged students and during the early period of vaccine availability for all elementary school-aged children. It is possible that findings would be different during different pandemic periods and with different conditions. Third, because case rate and hospitalization data are collected by decile of age group, we are not able to differentiate between case rates and hospitalization rates in 0–4-year-olds, who are too young to be attending primary and secondary school, and 5–9-year-olds, who do attend school. This limitation is particularly important for the hospitalization outcome variable, as those under 6 months of age are at substantially higher risk of severe SARS-CoV-2 than older children and thus results presented for the 0–9 year old age group may overestimate the impact of school masking policy de-adoption (37). Further, the inclusion of CDC data from individuals who are 19 years old and therefore unlikely to be a public-school student means that the 10–19 year old age group also includes individuals who were not in school. Fourth, there have been longitudinal increases in the use of home testing, resulting in decreasing publicly reportable test results, and in turn decreasing case ascertainment over time; however, during the study period, there was limited access to at-home testing, thus it is unlikely that this factor substantially impacted results. Fourth, there may be some misclassification of county-level vaccination rates, particularly for specific groups such as college students, who may be vaccinated at home but counted in census data where they attend school, or vise-versa. Individuals who split time between different counties may also have incorrect classification of their vaccination status. Finally, this study evaluated the real-world in-school masking policy changes as they were implemented. It did not assess compliance with the intervention. However, schools are a relatively controlled setting and thus compliance rates in schools were likely to be higher than in other parts of the community where there is less monitoring and feedback.

## Conclusion

Masking requirements were widely implemented during the pandemic as a mitigation measure designed to reduce SARS-CoV-2 cases in schools, with a secondary aim of limiting spread in the community. In this large retrospective cohort, we found that lifting of masking requirements in schools had, at most, minimal impacts in the event study design and no impact when the TTE framework was applied. These findings can be used to inform future pandemic response policies and risk–benefit discussions of mitigation measures implemented in elementary and secondary school settings, particularly when similar strategies are not applied in other settings.

## Research in context

**Evidence before this study:** Studies conducted prior to the COVID-19 pandemic suggested that schools might play a substantial role in spreading respiratory viruses in the community. Based on this limited evidence, mitigation policies were implemented across the United States with the aim of reducing spread in the surrounding community.

**Added value of this study:** This retrospective, observational study leverages two different causal inference approaches to measure the impact of the lifting of mask mandates in schools on key outcomes in the community. This study suggests that lifting of mask mandates in schools had little to no impact on key clinical outcomes, including hospitalizations and deaths.

**Implications of all of the available evidence:** Limited mitigation measures implemented in school settings had minimal or no impact on spread and key outcomes in the surrounding community. Findings can be used to inform policy decisions during future respiratory virus epidemics.

## Transparency statement

ZE and WB-E affirm that the manuscript is an honest, accurate, and transparent account of the study being reported, that no important aspects of the study have been omitted, and that any discrepancies from the study as originally planned have been explained.

## Copyright/license for publication

This work was done while employees of the federal government and the US federal government retains copyright.

## Data Availability

STATA code underlying the analysis are included in the Supplementary materials. Of note, the study includes data (the Burbio data) that are available for purchase. With permission from Burbio and confirmation of data use agreement with the CDC, datasets underlying the analysis will be shared upon written request to the authors.
