# Supplementary material for: Impact of lifting school mask mandates on community SARS-CoV-2 cases, hospitalizations, and deaths: a retrospective observational study
Source: Front Public Health. 2025 Jun 12;13:1579202. doi: 10.3389/fpubh.2025.1579202 (PMC12198242; doi:10.3389/fpubh.2025.1579202)
Supplement: Supplementary file 1 [file Data_Sheet_1.docx]

**Supplementary Table 1. Sources of Data**

| **Variable** | **Data Source** |
| --- | --- |
| Community incidence/hospitalization/death rate | CDC restricted access data |
| County population data | US Census Data  <https://www.census.gov/> |
| Student asymptomatic testing policy | Burbio Dataset   - Universal: All (required) - Partial: Opt-in, metric dependent, varies by grade level, varies by grade level - Selected populations: sports/extracurriculars (all types), required for preK, K - Unvaccinated only: unvaccinated required - None: Non, no info, limited info available, at home test kits   Excluded from analysis: we did no exclude any of asymptomatic testing options but since we filtered masking policy to include only Y, N, and Partial policies, the “universal” option in the testing requirement excluded. |
| Student vaccination requirement policy | Burbio Dataset   - None/selected populations= none, athletes and extracurriculars, athletes - Eligible populations = all eligible, all 12 and above - High school limited = all 16 and above   Excluded from analysis: N/A |
| Student masking policy | Burbio Dataset  Y: Fully required  Partial: Partially required  N: Not required  Excluded from the analysis: Y (metrics), N (state), Y (state), U, N (metrics), Partial (metrics), VC, MR, and No Info Available |
| Demographics (Race, ethnicity, poverty level) | CDC  ephtracking.cdc.gov |
| Urban/Rural | RUCA (<https://www.ers.usda.gov/data-products/rural-urban-commuting-area-codes/>) |
| Vaccination (fully vaccinated percentage)- stratified by age | CDC vaccination data |
| Mobility Movements | Google mobility data (Google COVID-19 Community Mobility Reports) |

**Supplementary Table 2.** COVID-19 Cases among 0-9 and 10-19 year olds per 100,000 county residents, estimated with a 2 week lag

|  | **Age Group 0-9** | | | **Age Group 10-19** | | |
| --- | --- | --- | --- | --- | --- | --- |
| **Cases per 100,000** | **Coefficient** | **95% Confidence** | **Interval** | **Coefficient** | **95% Confidence** | **Interval** |
| Fully vaccinated | -0.001** | -0.001 | -0.001 | -0.001** | -0.001 | -0.001 |
| Student Asymptomatic Testing  (Baseline: None) |  |  |  |  |  |  |
| - Unvaccinated Only | 79.71** | 42.21 | 117.21 | 193.73** | 127.73 | 259.73 |
| - Partial | 46.06** | 35.19 | 56.94 | 29.00** | 17.52 | 40.48 |
| - Selected populations | 72.57** | 33.61 | 111.54 | 61.61** | 20.00 | 103.21 |
| Student Vaccination Requirement  (Baseline: None/selected populations) |  |  |  |  |  |  |
| - High School Limited | N/A | N/A | N/A | 240.49** | 215.00 | 265.99 |
| - Eligible populations | -43.63** | -59.60 | -27.66 | -7.41 | -92.63 | 77.81 |
| Black | -1.85** | -2.10 | -1.60 | -2.39** | -2.67 | -2.10 |
| Other | -2.95* | -5.76 | -0.13 | -5.52** | -9.18 | -1.85 |
| American Indian | 0.10 | -0.44 | 0.64 | 0.56 | -0.18 | 1.29 |
| Poverty Level below 100% | 2.84** | 2.06 | 3.63 | 3.04** | 2.20 | 3.88 |
| Weeks from Policy Change  (Baseline: Week 0) |  |  |  |  |  |  |
| -4 | 0.99 | -3.28 | 5.26 | -3.22 | -7.82 | 1.39 |
| -3 | 45.95** | 35.22 | 56.67 | 36.90** | 25.40 | 48.40 |
| -2 | 51.04** | 41.18 | 60.90 | 36.04** | 25.70 | 46.38 |
| -1 | 35.72** | 26.84 | 44.60 | 16.07** | 7.16 | 24.98 |
| 1 | 9.55** | 4.89 | 14.21 | 5.95* | 0.95 | 10.96 |
| 2 | 8.70** | 4.25 | 13.15 | 3.55 | -1.21 | 8.31 |
| 3 | 16.69** | 12.07 | 21.32 | 10.96** | 5.99 | 15.93 |
| 4 | 22.68** | 17.88 | 27.47 | 18.26** | 12.97 | 23.55 |
| 5 | 26.51** | 21.66 | 31.37 | 21.56** | 16.32 | 26.80 |
| 6 | 27.28** | 22.64 | 31.91 | 22.19** | 17.08 | 27.31 |
| 7 | 27.34** | 22.74 | 31.95 | 23.46** | 18.38 | 28.54 |
| 8 | 40.58** | 36.08 | 45.07 | 38.76** | 33.61 | 43.92 |

** p<0.01, * p<0.05, Bolded p<0.1. Week 0 is the week when the masking policy was lifted in the district and results are stratified by decade of age. Results were generated from event-based regression 4 weeks before and 8 weeks after the policy changed and a 2-week lag.

**Supplementary Table 3**. COVID-19 Cases among 20-49 and 50-69, and 70+ year olds per 100,000 residents, estimated with a 2-week lag**.**

|  | **Age Group 20-49** | | | **Age Group 50-69** | | | **Age Group 70+** | | |
| --- | --- | --- | --- | --- | --- | --- | --- | --- | --- |
| **Cases per 100,000** | **Coefficient** | **95%**  **Confidence** | **Interval** | **Coefficient** | **95%**  **Confidence** | **Interval** | **Coefficient** | **95%**  **Confidence** | **Interval** |
| Fully vaccinated | -0.0001** | -0.0001 | -0.0001 | -0.0001** | -0.0001 | -0.00005 | -45.73* | -84.84 | -6.63 |
| Student Asymptomatic Testing  (Baseline: None) |  |  |  |  |  |  |  |  |  |
| - Unvaccinated Only | 263.38** | 204.59 | 322.16 | 140.97** | 93.27 | 188.67 | -25.88** | -39.34 | -12.41 |
| - Partial | 42.38** | 14.30 | 70.45 | **14.32** | -1.51 | 30.15 | -0.85 | -10.26 | 8.55 |
| - Selected populations | 130.88** | 35.20 | 226.56 | 35.90 | -12.95 | 84.75 | 1.04 | -26.41 | 28.49 |
| Student Vaccination Requirement  (Baseline: None/selected populations) |  |  |  |  |  |  |  |  |  |
| - High School Limited | 286.85** | 247.27 | 326.43 | 176.56** | 153.23 | 199.89 | -27.89** | -34.03 | -21.75 |
| - Eligible populations | 27.68 | -112.67 | 168.02 | -0.59 | -65.66 | 64.49 | -39.11** | -52.72 | -25.51 |
| Black | -6.53** | -7.31 | -5.74 | -4.13** | -4.59 | -3.67 | -2.92** | -3.19 | -2.65 |
| Other | -3.44 | -13.05 | 6.17 | -9.69** | -15.27 | -4.12 | -12.43** | -15.43 | -9.44 |
| American Indian | 3.14** | 1.06 | 5.21 | 2.34** | 1.13 | 3.55 | 0.73** | 0.20 | 1.26 |
| Poverty Level below 100% | 6.38** | 4.08 | 8.67 | 4.96** | 3.60 | 6.32 | 4.18** | 3.43 | 4.93 |
| Weeks from Policy Change  (Baseline: Week 0) |  |  |  |  |  |  |  |  |  |
| -4 | -7.43 | -20.50 | 5.64 | -2.03 | -9.68 | 5.63 | -2.33 | -6.72 | 2.07 |
| -3 | 99.72** | 68.38 | 131.07 | 64.17** | 45.51 | 82.83 | 35.61** | 24.73 | 46.50 |
| -2 | 110.19** | 81.88 | 138.51 | 70.34** | 53.15 | 87.54 | 43.99** | 34.35 | 53.63 |
| -1 | 59.51** | 35.83 | 83.19 | 37.40** | 22.91 | 51.90 | 21.29** | 12.90 | 29.68 |
| 1 | 27.28** | 14.69 | 39.87 | 10.18** | 2.72 | 17.63 | **4.13** | -0.42 | 8.69 |
| 2 | 32.68** | 20.39 | 44.98 | 14.78** | 7.45 | 22.11 | 6.32** | 1.98 | 10.65 |
| 3 | 44.28** | 31.41 | 57.14 | 26.27** | 18.79 | 33.75 | 12.52** | 8.08 | 16.95 |
| 4 | 66.48** | 52.99 | 79.97 | 38.74** | 30.86 | 46.61 | 16.64** | 12.07 | 21.21 |
| 5 | 71.88** | 58.76 | 85.00 | 48.91** | 40.94 | 56.89 | 18.49** | 13.98 | 23.00 |
| 6 | 80.98*** | 68.21 | 93.75 | 53.51** | 45.81 | 61.21 | 22.27** | 17.83 | 26.70 |
| 7 | 81.60** | 68.95 | 94.25 | 55.39** | 47.78 | 63.00 | 23.05** | 18.75 | 27.36 |
| 8 | 123.56** | 109.80 | 137.33 | 78.71** | 71.03 | 86.38 | 44.04** | 39.75 | 48.33 |

** p<0.01, * p<0.05, Bolded p<0.1Week 0 is the week when the masking requirement was lifted in schools, and results are stratified by decade of age. Results were generated from event- based regression 4 weeks before and 8 weeks after the policy changed with a 2-week lag.

**Supplementary Table 4.** COVID-19 olds per 100,000 residents, estimated with a 0-week lag**.**

| **Cases per 100,000** | **Whole cohort** | **0-9 Year olds** | **10-19 Year olds** | **20-49 Years olds** | **50-69 Year olds** | **70+ Year Olds** |
| --- | --- | --- | --- | --- | --- | --- |
| Fully vaccinated | **-3.74  [-5.02, -2.45]** | **-0.00004  [-0.00004, -0.00003]** | **-0.00002  [-0.00002, -0.00002]** | **-0.000003  [-0.000003, -0.000002]** | **-0.000002  [-0.000002, -0.000002]** | **-6.48  [-9.05, -3.91]** |
| Student Asymptomatic Testing  (Baseline: None) |  |  |  |  |  |  |
| - Unvaccinated Only | **-3.35  [-4.21, -2.5]** | 0.22  [-0.08, 0.51] | -0.11  [-0.43, 0.21] | **2.92  [1.34, 4.51]** | **2.46  [1.45, 3.47]** | **-2.29  [-3.83, -0.75]** |
| - Partial | **-0.67  [-0.97, -0.37]** | 0.24  [-0.37, 0.85] | -0.23  [-1.08, 0.62] | **-0.95  [-1.73, -0.18]** | **-0.67  [-1.17, -0.18]** | **-0.94  [-1.27, -0.62]** |
| - Selected populations | **-1.22  [-1.98, -0.47]** |  |  |  |  |  |
| Student Vaccination Requirement  (Baseline: None/selected populations) |  |  |  |  |  |  |
| - High School Limited | **-5.09  [-5.42, -4.77]** | **0.31  [0.02, 0.6]** | -0.22  [-0.59, 0.15] | **1.32  [0.13, 2.5]** | **1.32  [0.56, 2.08]** | **-4.43  [-4.77, -4.08]** |
| - Eligible populations | **-1.78  [-2.41, -1.15]** |  |  |  |  |  |
| Black | **-0.04  [-0.05, -0.03]** | **-0.02  [-0.03, -0.01]** | **-0.02  [-0.03, -0.01]** | **-0.05  [-0.08, -0.02]** | **-0.05  [-0.06, -0.03]** | **-0.05  [-0.07, -0.04]** |
| Other | **-0.87  [-1.02, -0.72]** | **-0.58  [-0.77, -0.39]** | **-0.61  [-0.86, -0.36]** | **-1.14  [-1.6, -0.69]** | **-1.01  [-1.28, -0.73]** | **-0.76  [-0.9, -0.62]** |
| American Indian | **0.08  [0.05, 0.11]** | **0.02  [0.01, 0.04]** | **0.04  [0.01, 0.07]** | **0.08  [0.01, 0.16]** | **0.07  [0.03, 0.12]** | 0.03  [0, 0.05] |
| Poverty Level below 100% | **-0.11  [-0.14, -0.08]** | **-0.11  [-0.15, -0.08]** | **-0.11  [-0.15, -0.07]** | **-0.28  [-0.37, -0.19]** | **-0.17  [-0.23, -0.11]** | -0.02  [-0.06, 0.01] |
| Weeks from Policy Change  (Baseline: Week 0) |  |  |  |  |  |  |
| -4 | **1.24  [0.92, 1.57]** | **0.58  [0.18, 0.97]** | **0.67  [0.26, 1.08]** | **1.48  [0.44, 2.51]** | **1.03  [0.43, 1.62]** | **1.46  [1.08, 1.84]** |
| -3 | **0.67  [0.4, 0.94]** | **0.68  [0.29, 1.07]** | 0.23  [-0.16, 0.62] | 0.63  [-0.19, 1.44] | **0.81  [0.25, 1.37]** | **0.83  [0.43, 1.23]** |
| -2 | 0.02  [-0.25, 0.28] | 0.28  [-0.03, 0.59] | 0.19  [-0.19, 0.56] | 0.04  [-0.82, 0.91] | 0.07  [-0.5, 0.64] | 0.36  [-0.01, 0.73] |
| -1 | 0.14  [-0.04, 0.33] | 0.11  [-0.11, 0.34] | 0.11  [-0.15, 0.37] | 0.06  [-0.56, 0.67] | 0.06  [-0.33, 0.45] | 0.19  [-0.09, 0.47] |
| 1 | **-0.6  [-0.81, -0.4]** | **-0.35  [-0.6, -0.1]** | **-0.34  [-0.57, -0.11]** | **-0.91  [-1.53, -0.28]** | **-0.77  [-1.18, -0.36]** | **-0.63  [-0.95, -0.32]** |
| 2 | **-0.85  [-1.11, -0.59]** | **-0.42  [-0.74, -0.1]** | -0.09  [-0.43, 0.25] | **-0.83  [-1.65, -0.01]** | **-0.71  [-1.21, -0.21]** | **-0.83  [-1.16, -0.51]** |
| 3 | **-0.91  [-1.18, -0.64]** | **-0.48  [-0.83, -0.13]** | -0.17  [-0.52, 0.18] | -0.84  [-1.69, 0.02] | -0.7  [-1.21, -0.19] | -0.83  [-1.17, -0.5] |
| 4 | **-0.7  [-0.98, -0.42]** | -0.3  [-0.66, 0.05] | 0.09  [-0.28, 0.46] | -0.51  [-1.39, 0.38] | -0.47  [-1.01, 0.06] | -0.75  [-1.1, -0.4] |
| 5 | **-0.74  [-1.02, -0.45]** | -0.29  [-0.65, 0.07] | -0.15  [-0.52, 0.22] | -0.65  [-1.58, 0.27] | -0.31  [-0.88, 0.25] | **-0.58  [-0.93, -0.22]** |
| 6 | **-0.33  [-0.64, -0.02]** | 0.02  [-0.36, 0.4] | 0.2  [-0.18, 0.58] | -0.04  [-1.05, 0.98] | 0.28  [-0.33, 0.9] | -0.37  [-0.75, 0] |
| 7 | **-0.67  [-0.97, -0.37]** | -0.23  [-0.61, 0.14] | -0.17  [-0.55, 0.22] | -0.85  [-1.83, 0.12] | -0.1  [-0.68, 0.48] | -0.49  [-0.86, -0.13] |
| 8 | **-1.17  [-1.47, -0.86]** | -0.33  [-0.7, 0.04] | -0.29  [-0.69, 0.11] | -1.87  [-2.85, -0.89] | -0.71  [-1.29, -0.12] | -0.68  [-1.04, -0.31] |

Variables in bold, p< 0.05.. Week 0 is the week when the masking policy was lifted in the district and results are stratified by decade of age. Results were generated from event-based regression 4 weeks before and 8 weeks after the policy changed and a no lag.

**Supplementary Table 5. Demographics of Excluded Counties***

|  | **Variable** | **All Excluded Counties** | **Excluded Counties with 1 District** |
| --- | --- | --- | --- |
|  |  | **Included Counties (%) / Included Districts (School Population)** | **Included Counties (%) / Included Districts (School Population)** |
| **Total School Population** |  | 28,429,871 | 6,956,216 |
| **Race*** | White | 83.11% | 82.39% |
|  | American Indian | 2.46% | 2.69% |
|  | Black | 8.79% | 10.31% |
|  | Other | 2.6% | 2.44% |
|  | Asian^€^ | 3.04% | 2.17% |
| **Poverty Level*** | Federal Poverty Level Less than 100% | 19.42% | 20.35% |
| **Region*** | Northeast | 4,675,714 | 63,282 |
|  | Midwest | 5,955,350 | 249,725 |
|  | South | 9,073,464 | 5,589,365 |
|  | West | 8,725,343 | 1,053,844 |
| **Urban/Rural** | Urban | 25,682,986 | 6,069,172 |
|  | Suburban | 2,205,140 | 654,760 |
|  | Rural | 541,745 | 232,284 |
| **Mobility** | Grocery | 2.12 | 3.49 |
|  | Park | 15.62 | 7.57 |
|  | Residential | 4.67 | 3.97 |
|  | Retail | -3.13 | -1.71 |
|  | Transit | -5.09 | -2.19 |
|  | Workplace | -21.86 | -20.55 |
| **Vaccination Uptake^*€^ (%)** | 5–11-year-old | 13.89% | 11.22% |
|  | 12–17-year-old | 43.87% | 38.86% |
|  | 18–64-year-old | 57.79% | 53.40% |
|  | > 65-year-old | 81.04% | 77.16% |
| **Other Non-Pharmaceutical Interventions in Schools** | Student Asymptomatic Testing Policy |  |  |
|  | - Partial | 7,491,812 | 1,581,175 |
|  | - Selected populations | 1,798,196 | 1,021,737 |
|  | - Unvaccinated only | 124,580 | 80,479 |
|  | - None | 24,347,030 | 6,367,669 |
|  | School Vaccination Policy |  |  |
|  | - None | 27,680,489 | 6,956,216 |
|  | - Eligible Population | 768,079 | 0 |
|  | - High School Limited | 546 | 0 |

*Excluded counties includes those that lifted their mask mandate prior to 10/28/2021.

**Supplementary Table 6**. Absolute incidence of cases, hospitalizations, and deaths among the control and intervention counties included in the target trial emulation framework analysis.

|  | **Intervention Counties**  **(In-School Mask Requirement Lifted)*** | | | **Control Counties**  **(In-School Mask Requirement Maintained)*** | | |
| --- | --- | --- | --- | --- | --- | --- |
| **Age Category** | **Cases** | **Hospitalizations** | **Deaths** | **Cases** | **Hospitalizations** | **Deaths** |
| **0-9 Year-olds** | 3211 | 104 | 6 | 13745 | 701 | 18 |
| **10-19 Year-olds** | 4063 | 113 | 20 | 15240 | 617 | 24 |
| **20-49 Year-olds** | 16367 | 1435 | 147 | 51202 | 5486 | 491 |
| **50-69 Year-olds** | 10738 | 2552 | 519 | 34463 | 8093 | 1132 |
| **≥ 70 Year-olds** | 6829 | 2892 | 696 | 24654 | 9200 | 1646 |

**Supplementary Table 7**. Unadjusted results of counties included in the Target Trial Emulation

|  |  | **Mask Mandate Maintained** | **Mask Mandate Lifted** | **Difference** | **95% Confidence**  **Interval** | | **P-value** |
| --- | --- | --- | --- | --- | --- | --- | --- |
| **Cases per 100,000** | **Whole Cohort** | **14.31** | **16.12** | **1.807** | **1.117** | **2.497** | **0.000** |
|  | *0-9 year olds* | 8.39 | 8.70 | 0.307 | -0.551 | 1.165 | 0.483 |
|  | *10-19 year olds* | 10.15 | 10.53 | 0.382 | -0.625 | 1.390 | 0.457 |
|  | *20-49 year olds* | 23.18 | 24.32 | 1.135 | -0.793 | 3.064 | 0.248 |
|  | *50-69 year olds* | **15.51** | **18.53** | **3.019** | **1.596** | **4.441** | **0.000** |
|  | *70+ year olds* | **10.29** | **12.81** | **2.520** | **1.406** | **3.634** | **0.000** |
|  |  |  |  |  |  |  |  |
| **Hospitalizations per 1,000,000** | **Whole Cohort** | **17.03** | **23.65** | **6.618** | **4.881** | **8.354** | **0.000** |
|  | *0-9 year olds* | 2.26 | 2.65 | 0.392 | -0.771 | 1.555 | 0.509 |
|  | *10-19 year olds* | 1.78 | 2.53 | 0.745 | -0.171 | 1.662 | 0.111 |
|  | *20-49 year olds* | **13.32** | **16.01** | **2.684** | **0.272** | **5.097** | **0.029** |
|  | *50-69 year olds* | **25.72** | **34.73** | **9.008** | **5.298** | **12.719** | **0.000** |
|  | *70+ year olds* | **33.97** | **47.77** | **13.801** | **8.082** | **19.520** | **0.000** |
| **Deaths per 1,000,000** | **Whole Cohort** | **6.35** | **11.11** | **4.753** | **3.514** | **5.993** | **0.000** |
|  | *0-9 year olds* | 0.08 | 0.09 | 0.009 | -0.144 | 0.163 | 0.904 |
|  | *10-19 year olds* | 0.10 | 0.34 | 0.237 | -0.021 | 0.495 | 0.072 |
|  | *20-49 year olds* | 2.56 | 3.35 | 0.784 | -0.174 | 1.741 | 0.109 |
|  | *50-69 year olds* | **8.90** | **15.33** | **6.425** | **4.056** | **8.794** | **0.000** |
|  | *70+ year olds* | **17.18** | **29.46** | **12.275** | **7.593** | **16.958** | **0.000** |

**Supplementary Table 8**. Target Trial Emulation Framework: Average Impact of Lifting of Mask Mandates in US Counties (Sensitivity analysis with follow-up extended to 8 weeks)

| **Cases per 100,000 County Residents** |  | **Mask Mandate Maintained** | **Mask Mandate Lifted** | **Difference*** | **95% Confidence**  **Interval** | | **P-value** |
| --- | --- | --- | --- | --- | --- | --- | --- |
|  | **Whole Cohort** | 12.4 | 4.31 | **-8.08** | **-15.65** | **-0.50** | **0.04** |
|  | **0-9 years old** | 6.04 | 2.02 | -4.03 | -8.48 | 0.42 | 0.08 |
|  | **10-19 years old** | 3.03 | 2.34 | -0.68 | -6.71 | 5.34 | 0.82 |
|  | **20-49 years old** | 14.30 | 7.74 | -6.56 | -24.58 | 11.46 | 0.47 |
|  | **50-69 years old** | 19.68 | 5.16 | -14.52 | -40.03 | 10.98 | 0.26 |
|  | **70+ years old** | 10.80 | 3.35 | -7.45 | 0.05 | -14.77 | -0.13 |
| **Hospitalizations per 1,000,000 County Residents** | **Whole Cohort** | 25.18 | 9.31 | -15.87 | -31.83 | 0.09 | >0.05 |
|  | **0-9 years old** | 0.52 | 1.21 | 0.69 | -0.14 | 1.52 | 0.11 |
|  | **10-19 years old** | 3.19 | 1.1 | -2.1 | -11.46 | 7.26 | 0.66 |
|  | **20-49 years old** | 11.11 | 8.69 | -2.42 | -16.15 | 11.3 | 0.73 |
|  | **50-69 years old** | 50.91 | 14.63 | -36.28 | -96.61 | 24.05 | 0.24 |
|  | **70+ years old** | 31.12 | 18.36 | -12.76 | -13.18 | 1.75 | 0.12 |
| **Deaths per 1,000,000 County Residents** | **Whole Cohort** | 6.49 | 3.93 | -2.56 | -6.08 | 0.97 | 0.16 |
|  | **0-9 years old** | 0.16 | 0.06 | -0.1 | -0.41 | 0.21 | 0.3 |
|  | **10-19 years old** | 0.14 | 0.25 | **0.11** | **0.08** | **0.28** | **0.05** |
|  | **20-49 years old** | 4.75 | 2.36 | -2.39 | 0.48 | -9.1 | 4.33 |
|  | **50-69 years old** | 11.05 | 6.10 | -4.95 | 0.34 | -15.13 | 5.24 |
|  | **70+ years old** | 15.57 | 9.72 | -5.85 | -16.01 | 4.32 | 0.26 |

*Difference in case rates. Variables in bold, p< 0.05.

**Supplementary Figure 1.** Unadjusted mean COVID-19 cases per 100,000 county residents per week among 0–9 and 10-19-year-olds during the study period


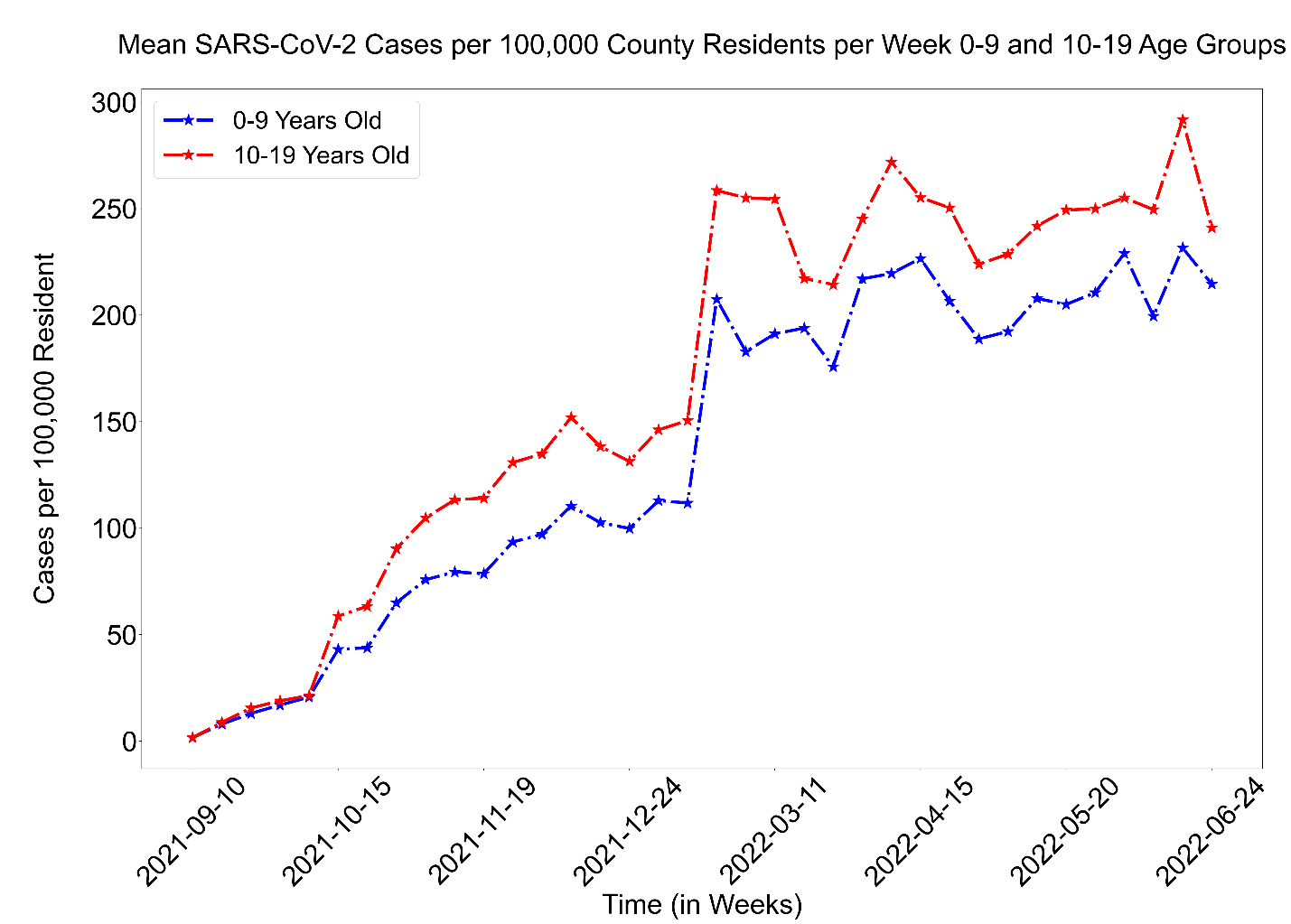


**Supplementary Figure 2.** Unadjusted mean COVID-19 cases per 100,000 county residents per week among 20-49, 50-69, 70+ year olds during the study period


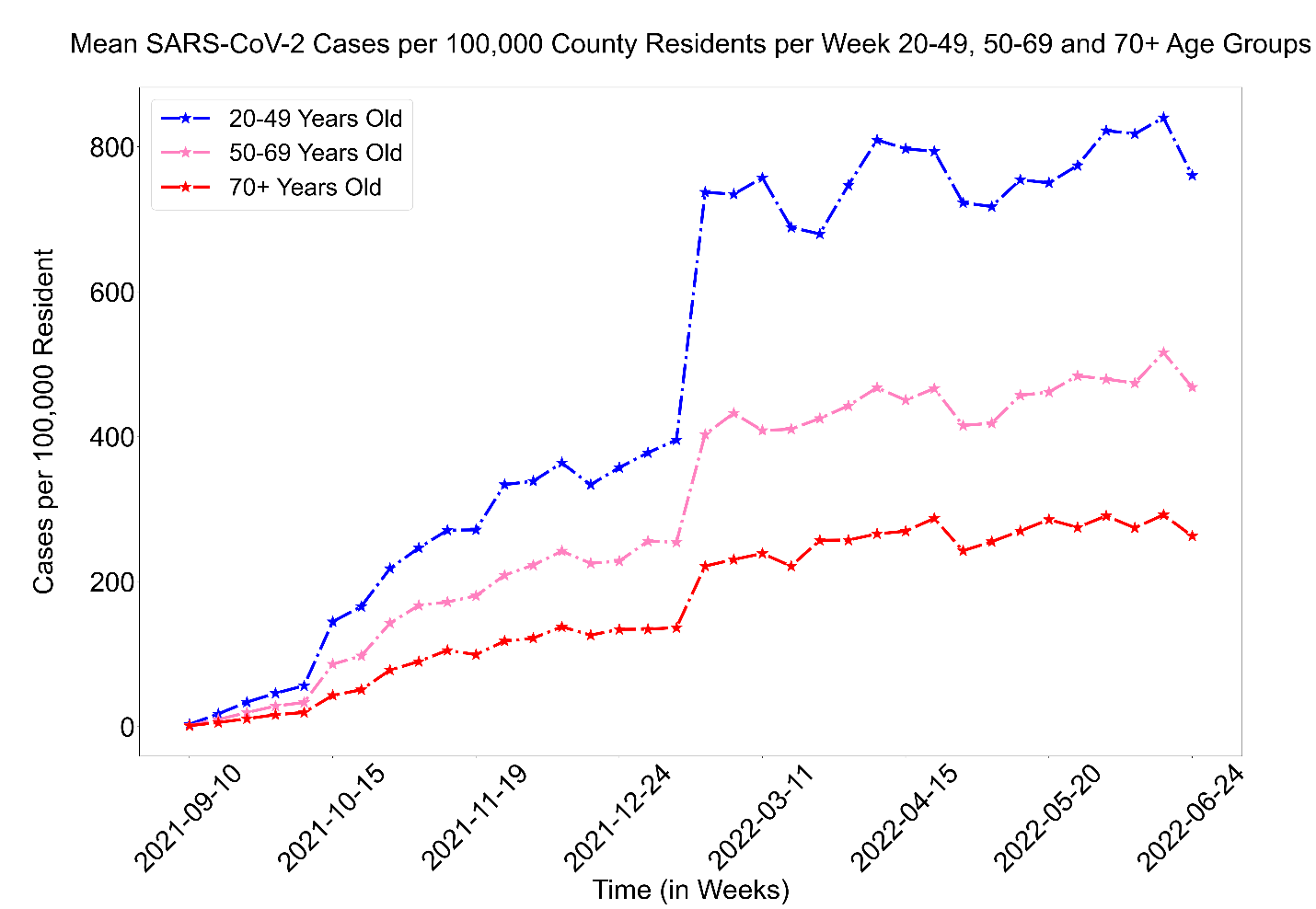


**Supplementary Figure 3.** Unadjusted hospitalizations per 1,000,000 county residents per week among 0-9 and 10-19 year olds during the study period


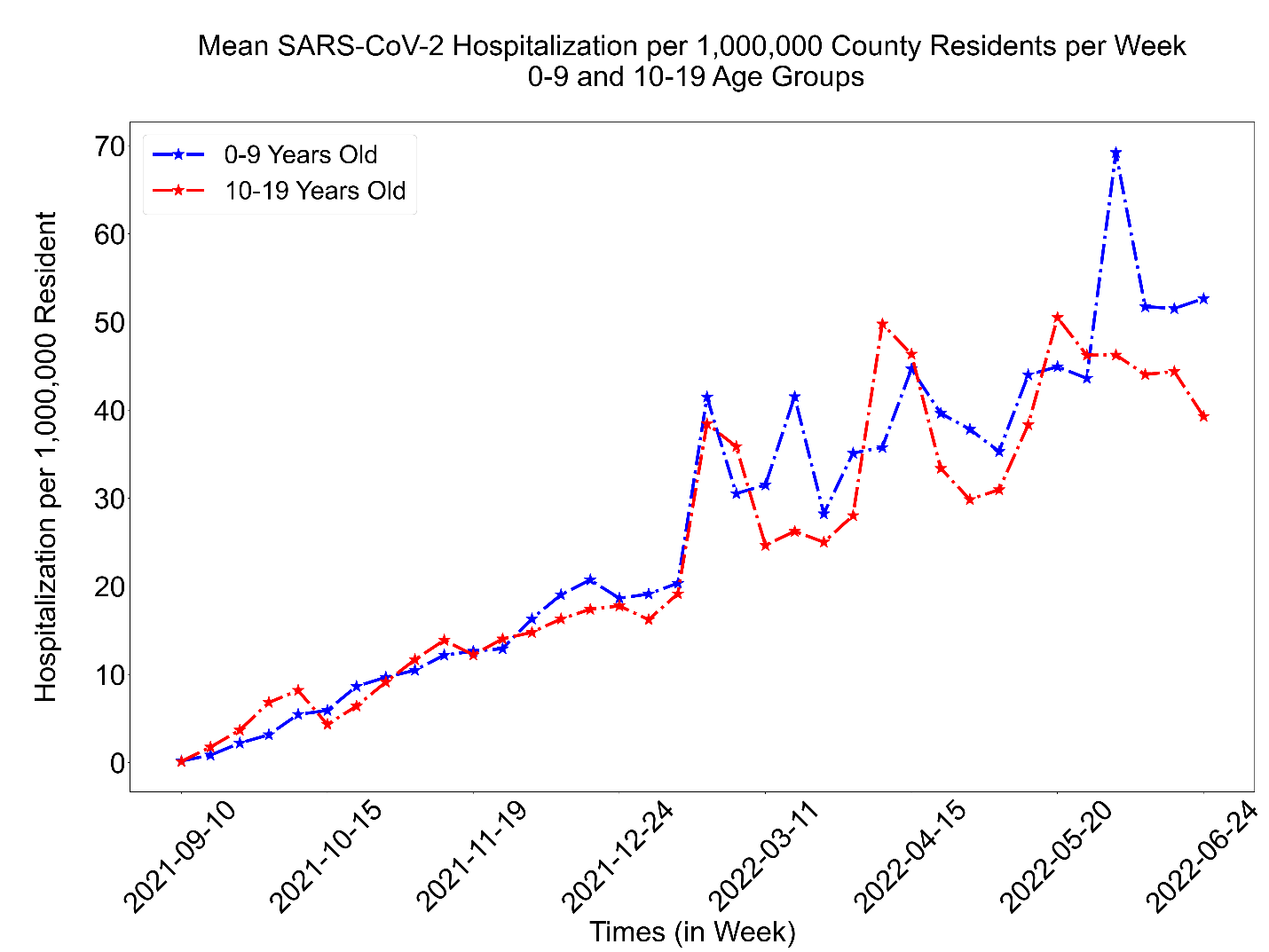


**Supplementary Figure 4.** Unadjusted hospitalizations per 1,000,000 county residents per week among 20-49, 50-69, and 70+ year olds during the study period


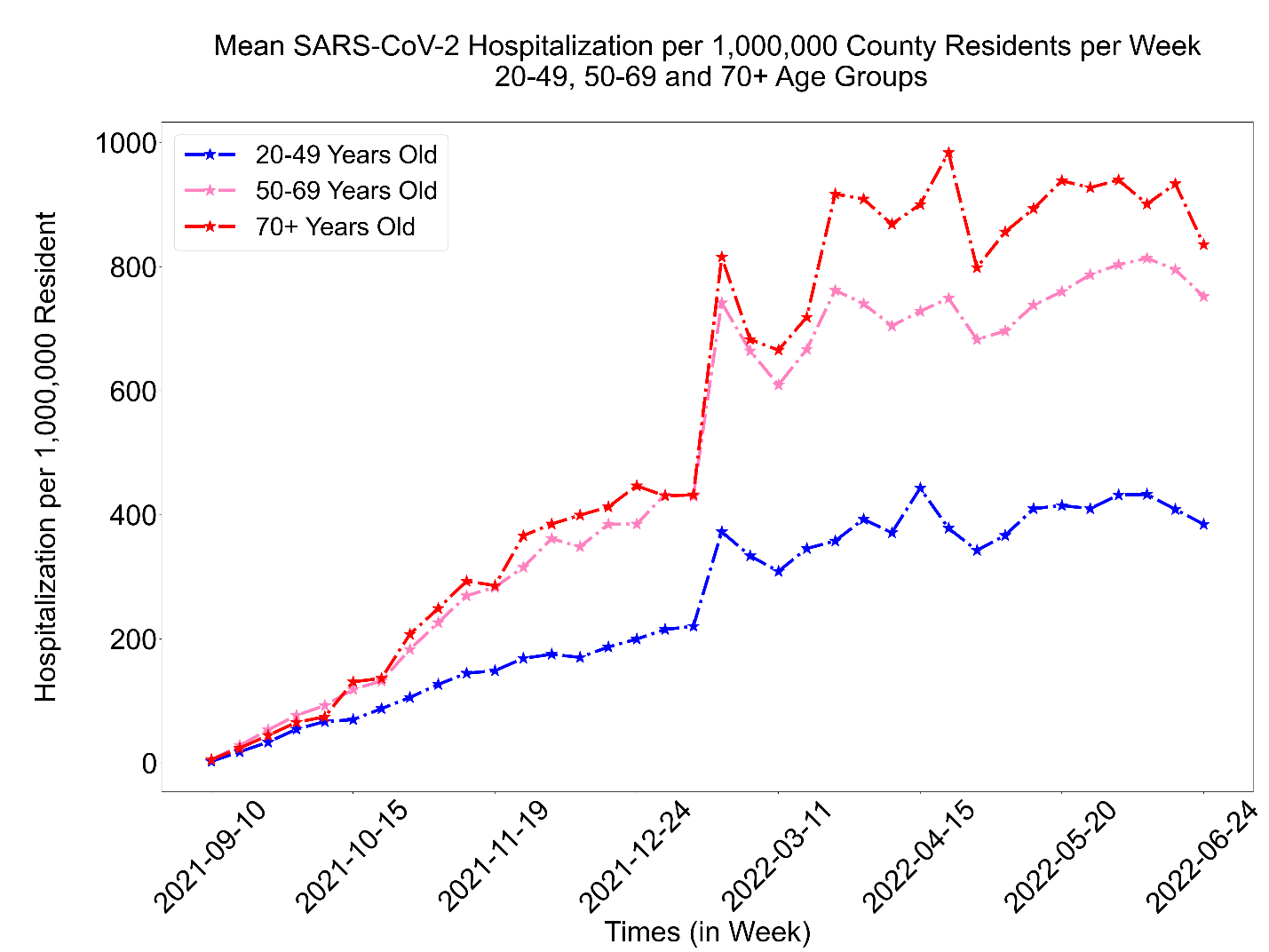


**Supplementary Figure 5.** Unadjusted deaths per 1,000,000 county residents per week among 0-9 and 10-19 year olds during the study period


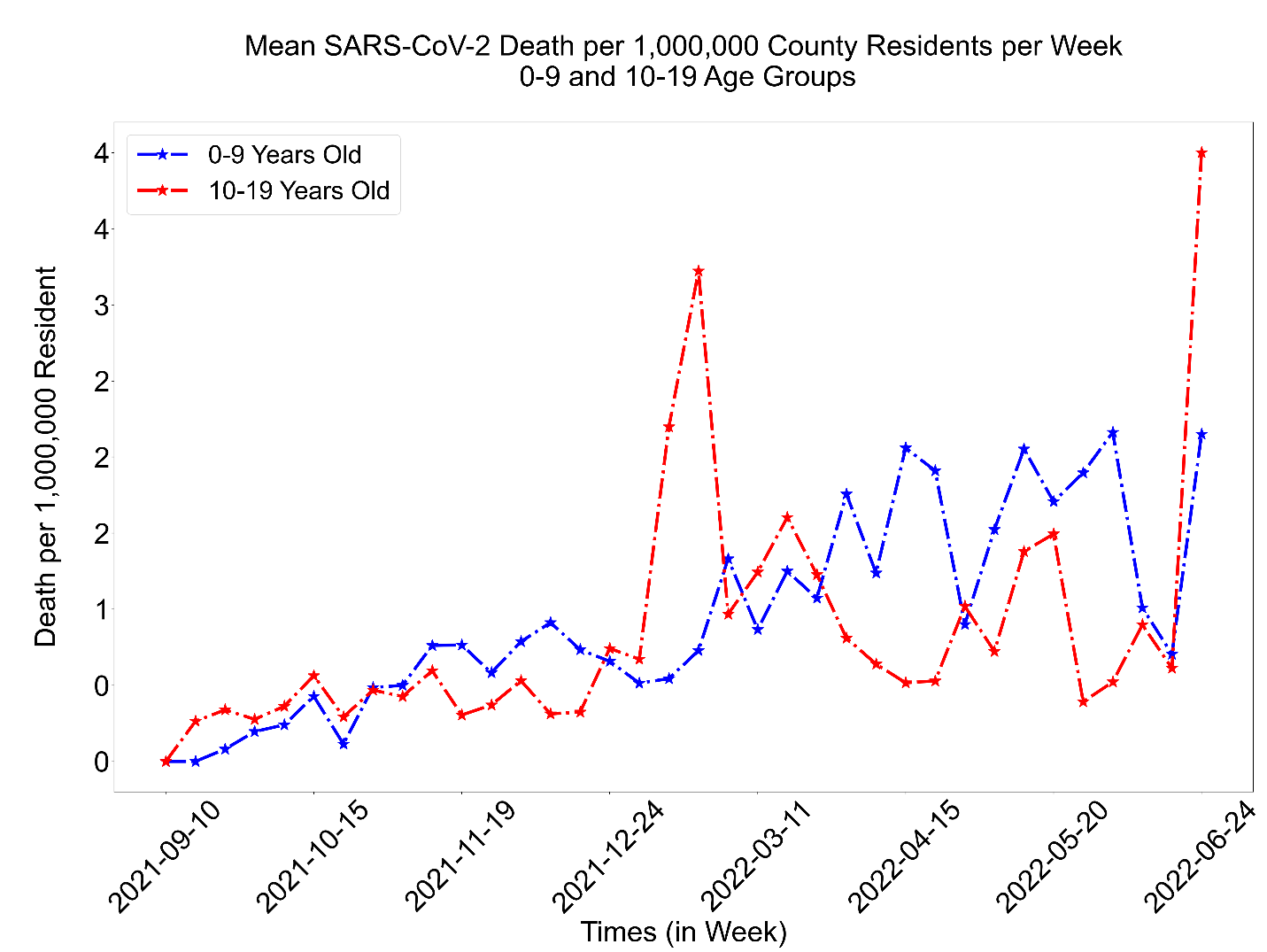


**Supplementary Figure 6.** Unadjusted deaths per 1,000,000 county residents per week among 20-49, 50-69, and 70+ year olds during the study period


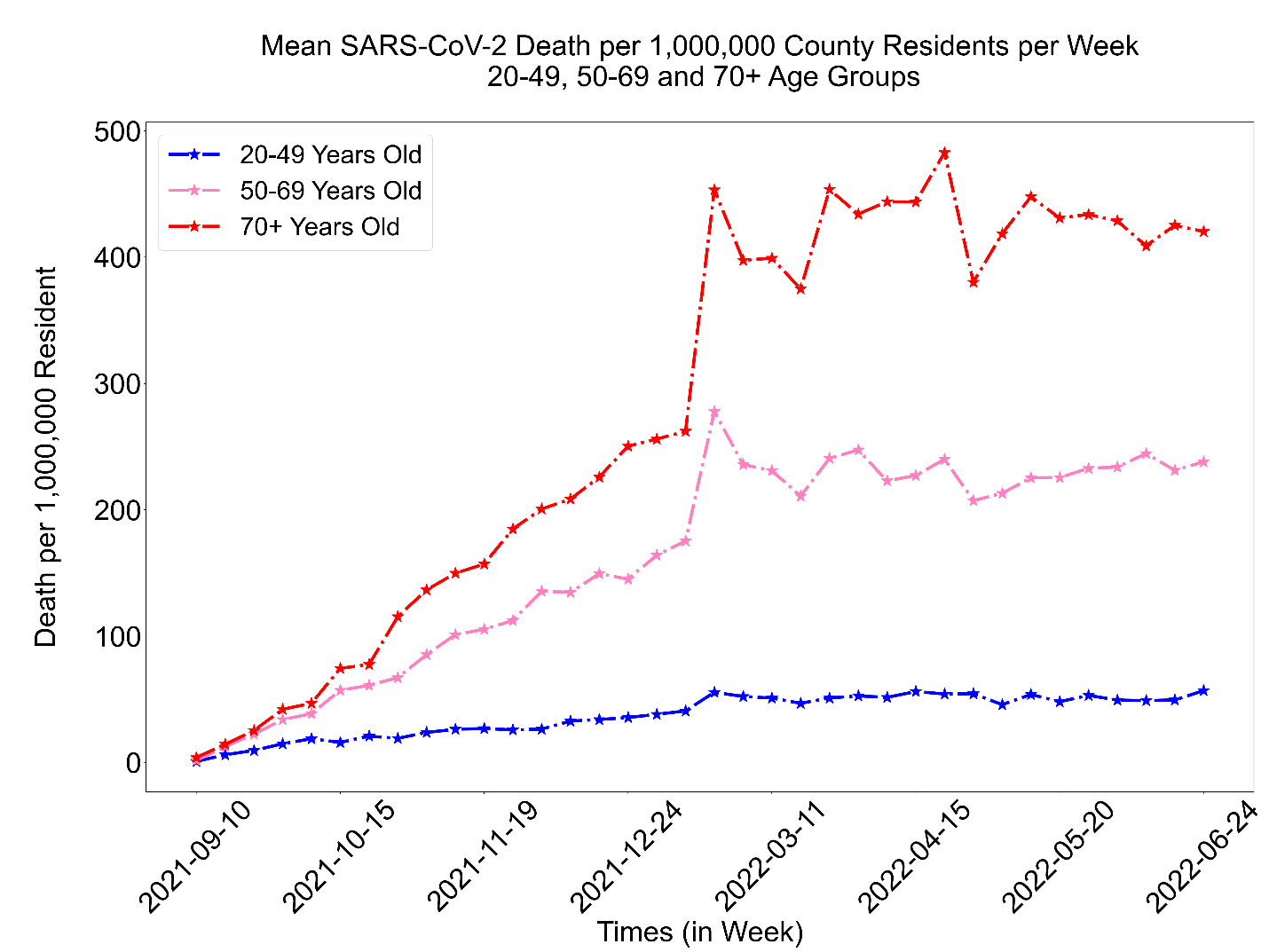


**Supplementary Figure 7.** Unadjusted mean case rates per 100,000 county residents and hospitalization and death rates per 1,000,000 county residents, 0-9 year olds, stratified by date of mask mandate lifting


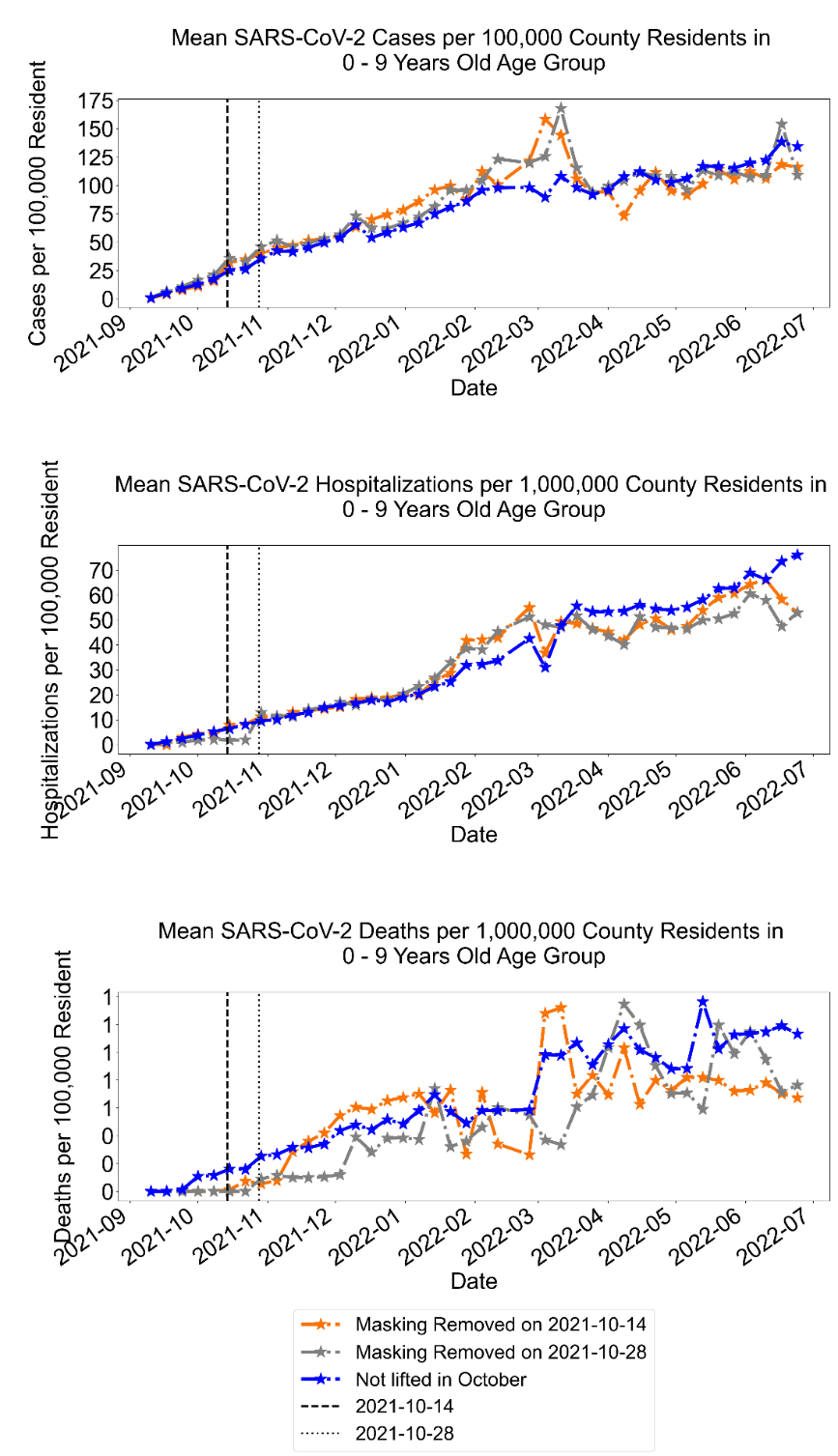


**Supplementary Figure 8.** Unadjusted mean case rates per 100,000 county residents and hospitalization and death rates per 1,000,000 county residents, 10-19 year olds, stratified by date of mask mandate lifting


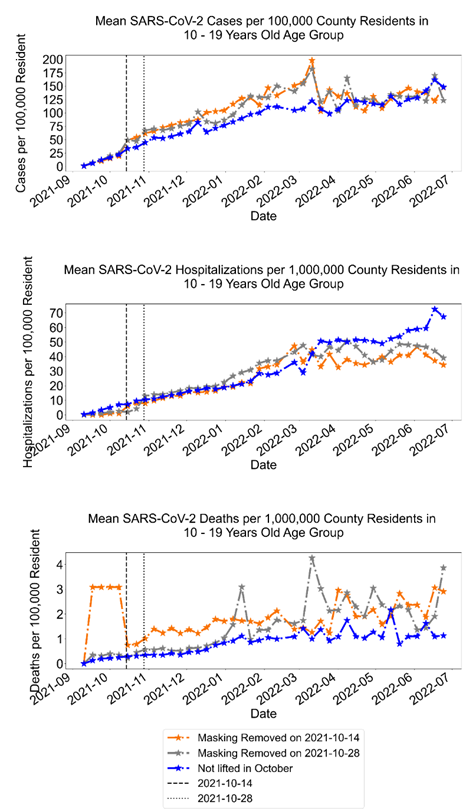


**Supplementary Figure 9.** Unadjusted mean case rates per 100,000 county residents and hospitalization and death rates per 1,000,000 county residents, 20-49 year olds, stratified by date of mask mandate lifting


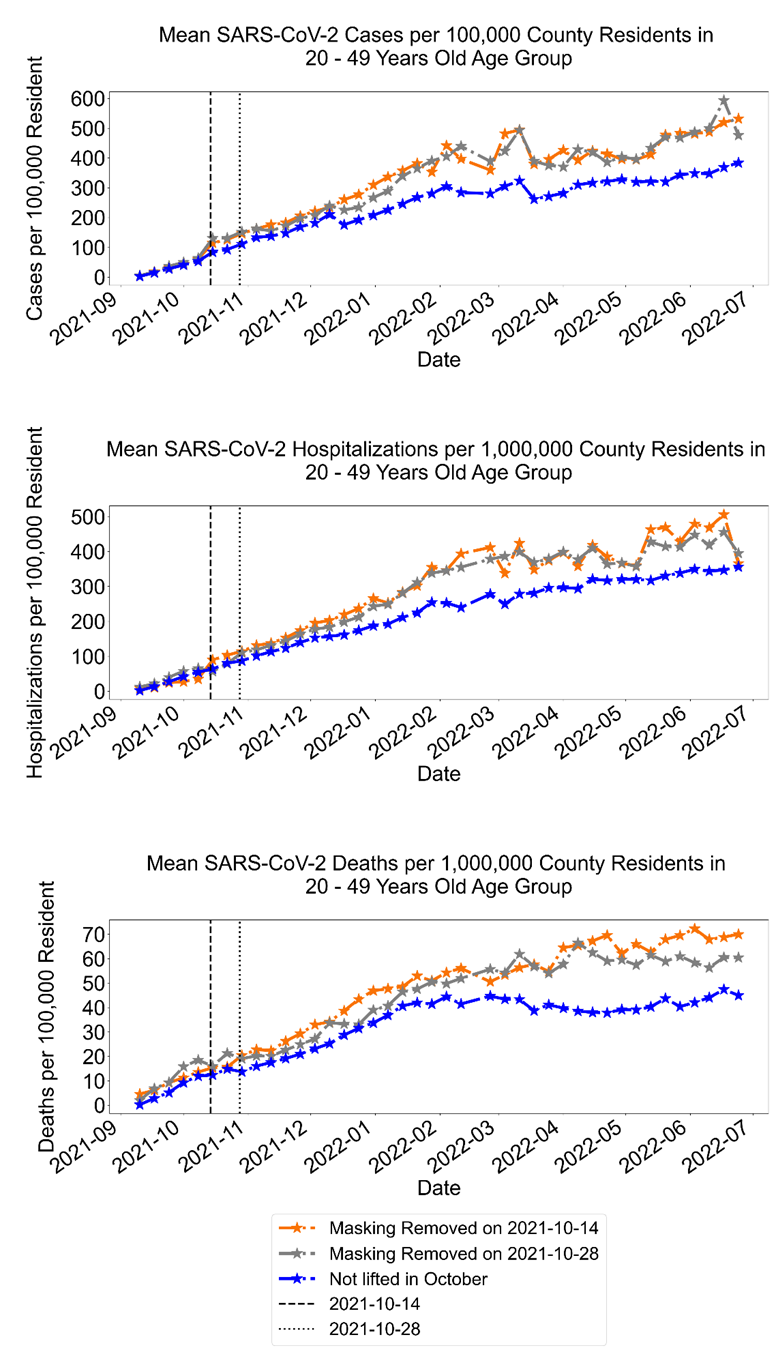


**Supplementary Figure 10.** Unadjusted mean case rates per 100,000 county residents and hospitalization and death rates per 1,000,000 county residents, 50-69 year olds, stratified by date of mask mandate lifting


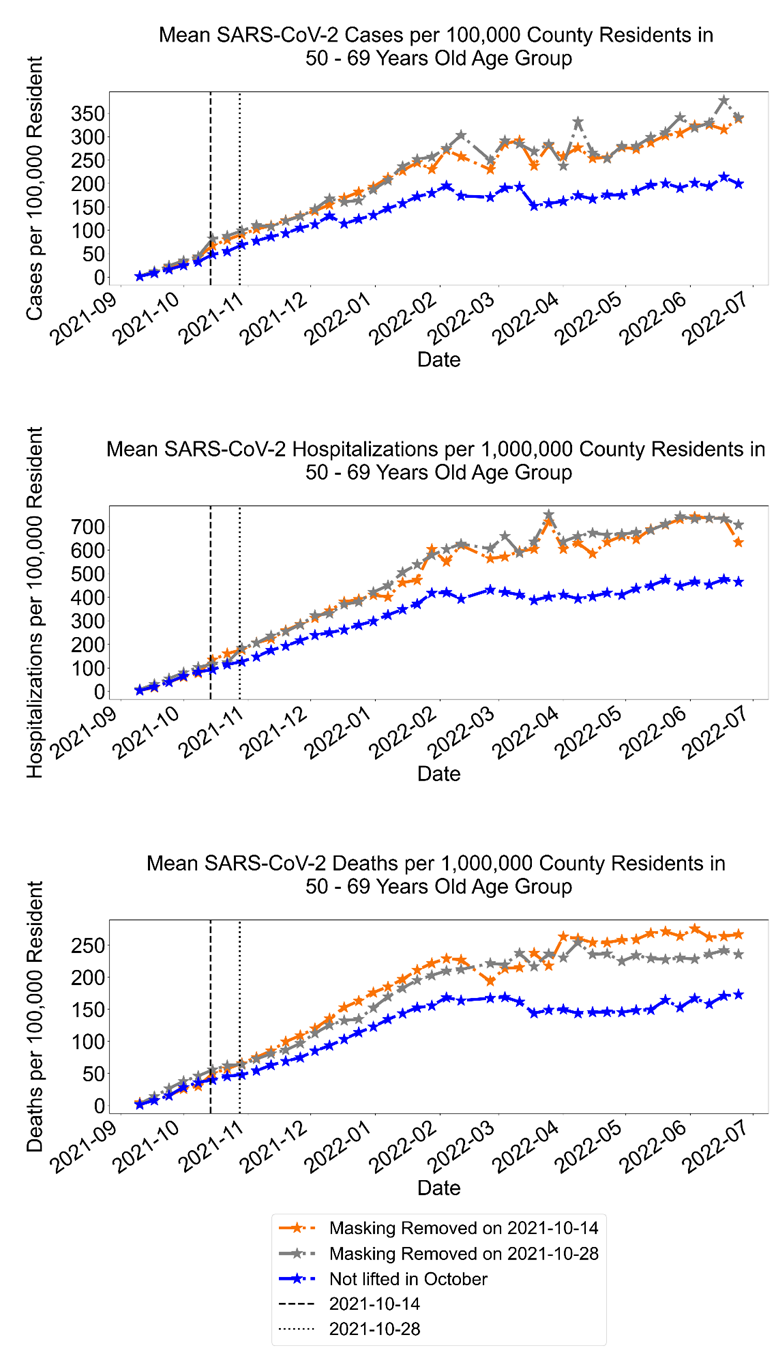


**Supplementary Figure 11.** Unadjusted mean case rates per 100,000 county residents and hospitalization and death rates per 1,000,000 county residents, 70+ year olds, stratified by date of mask mandate lifting


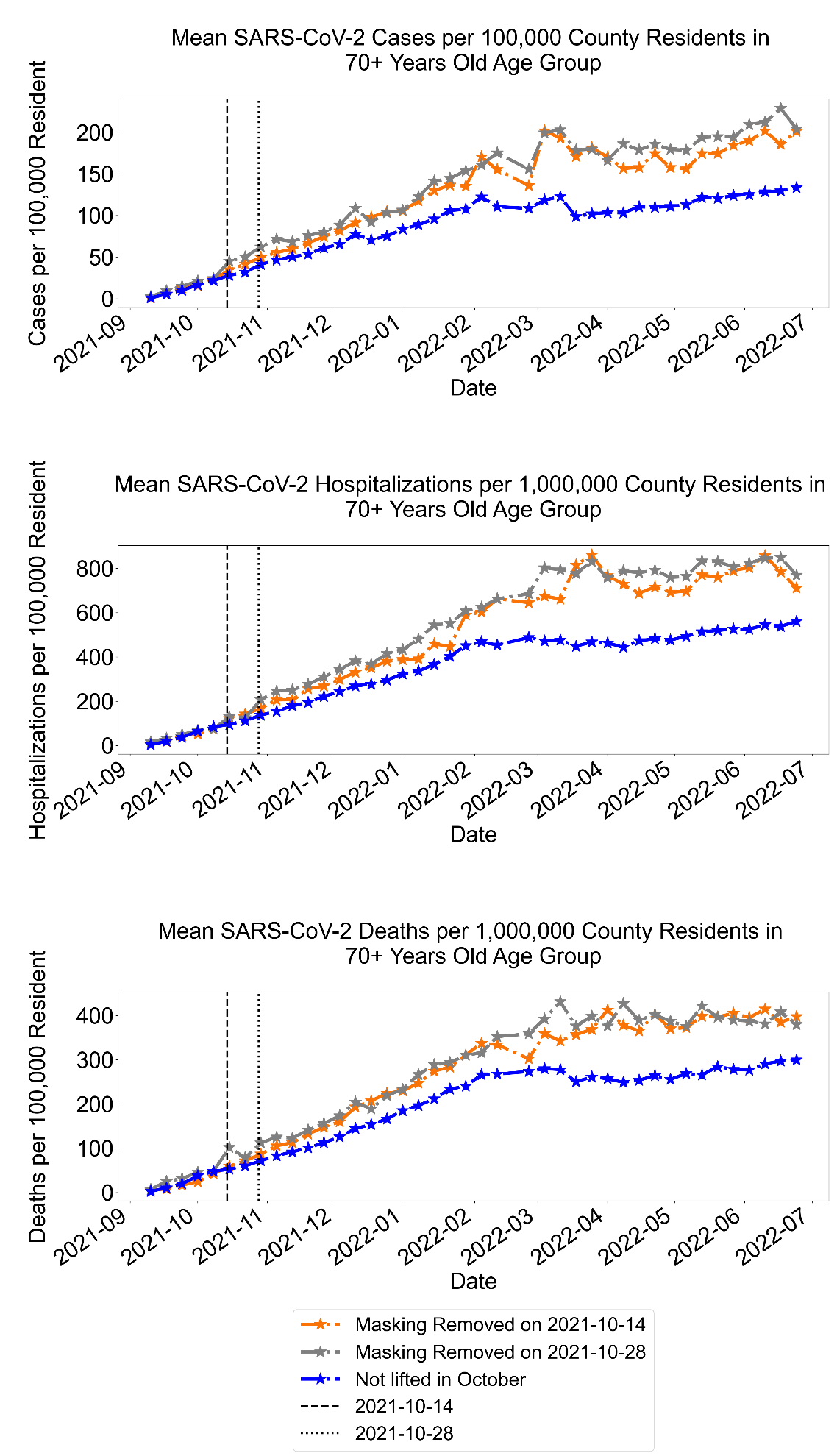


**Supplementary Figure 12.** Google mobility trends for retail and recreation, stratified by date of masking policy change.


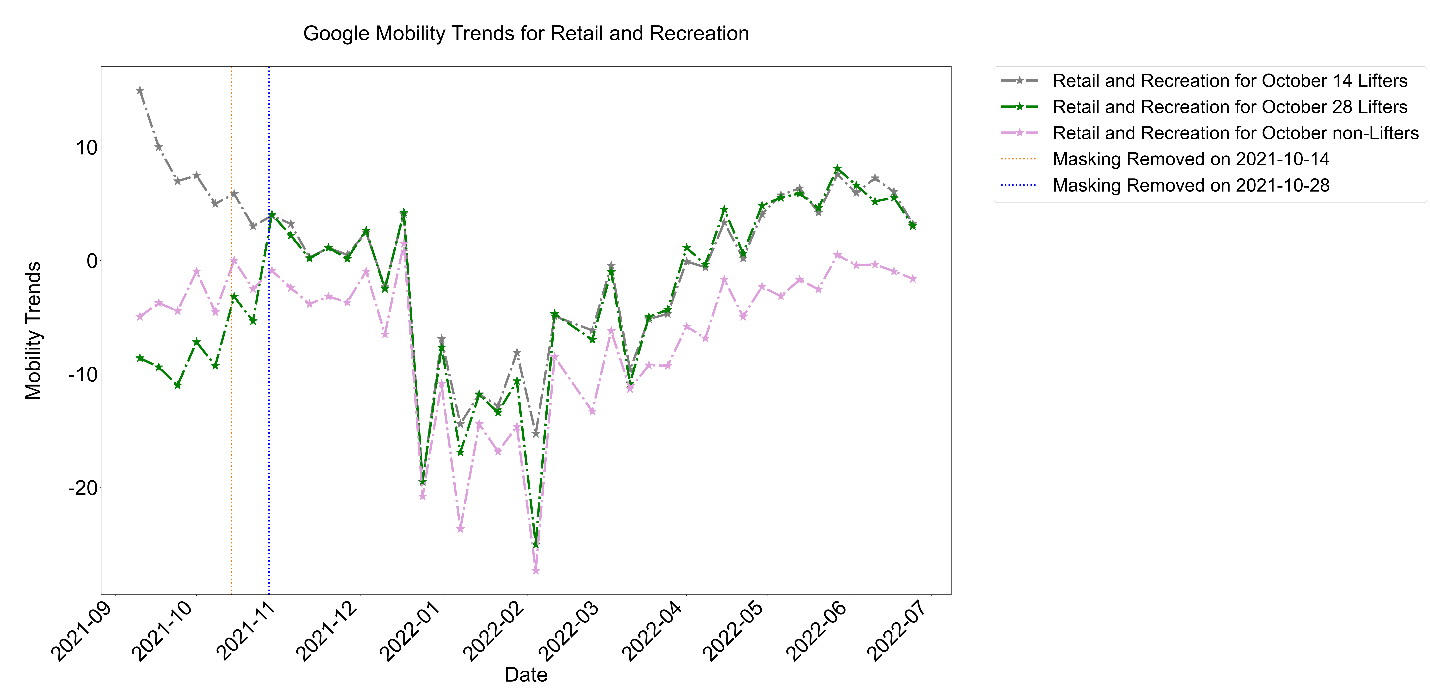


**Supplementary Figure 13**. Frequency of Removing Masking policy date in all counties


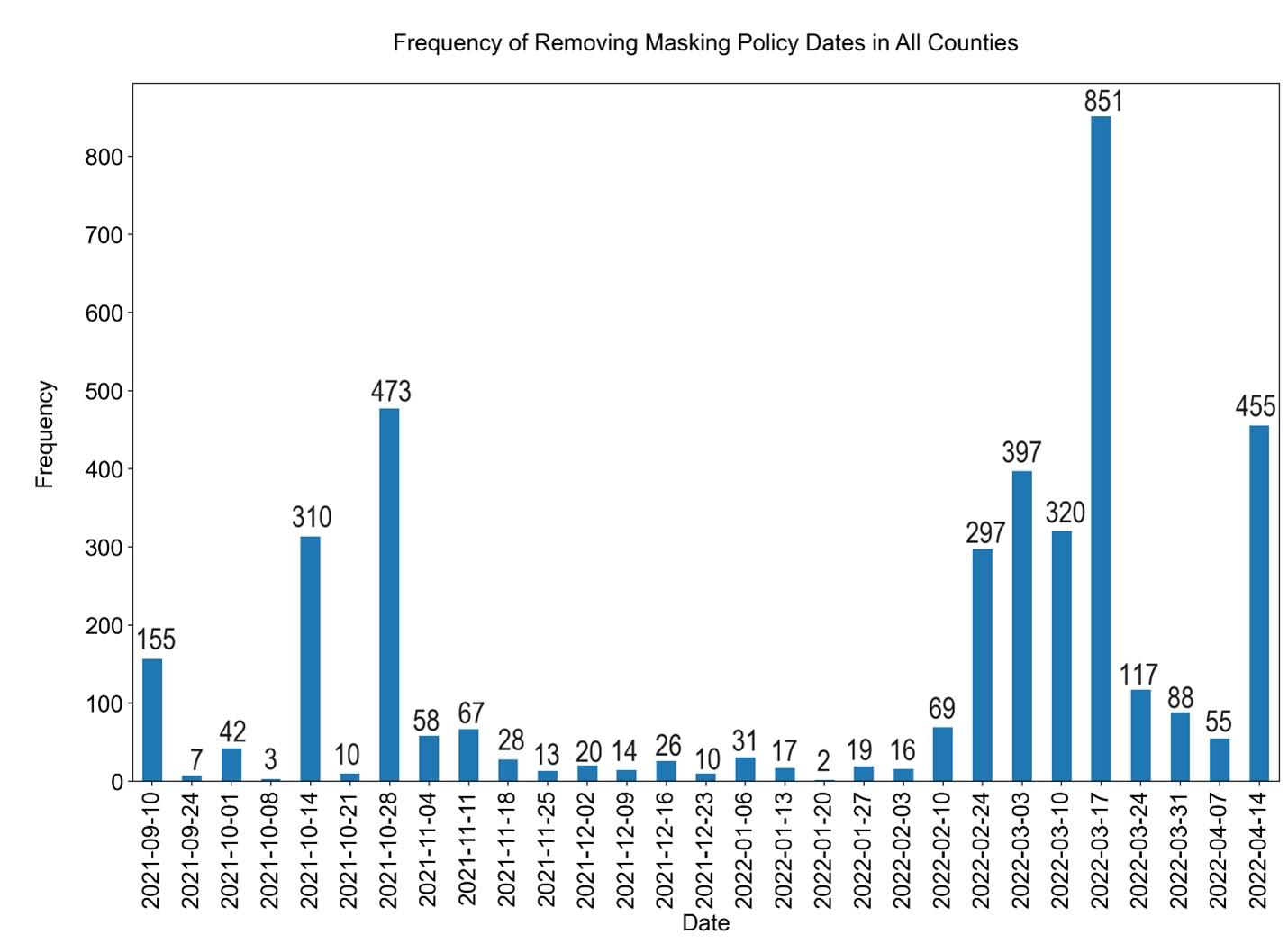


**Supplementary Figure 14**. Frequency of mask policy lifting date in counties with one school district


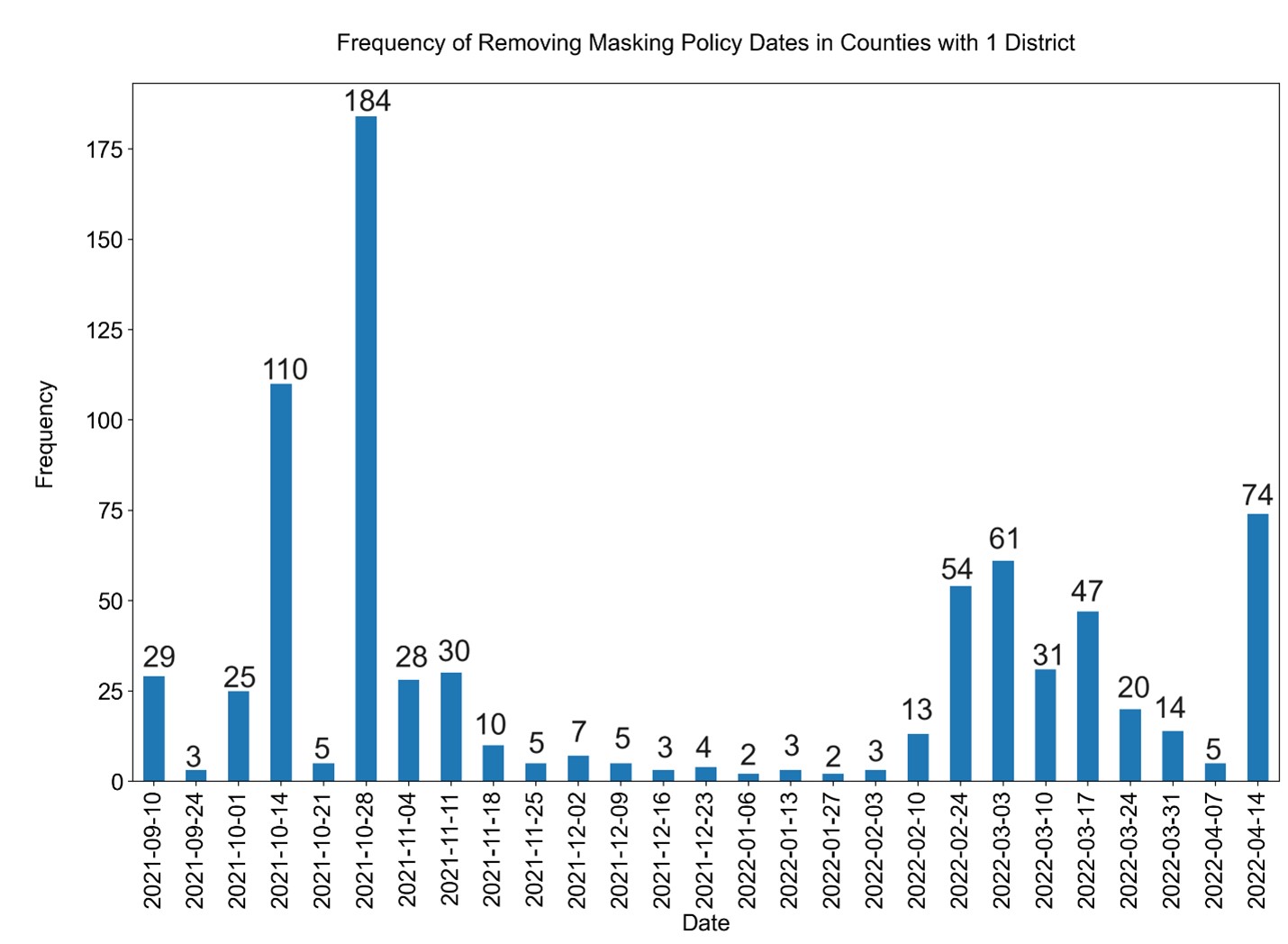


**Supplementary Figure 15**. Frequency of mask mandate removal per week among included counties.


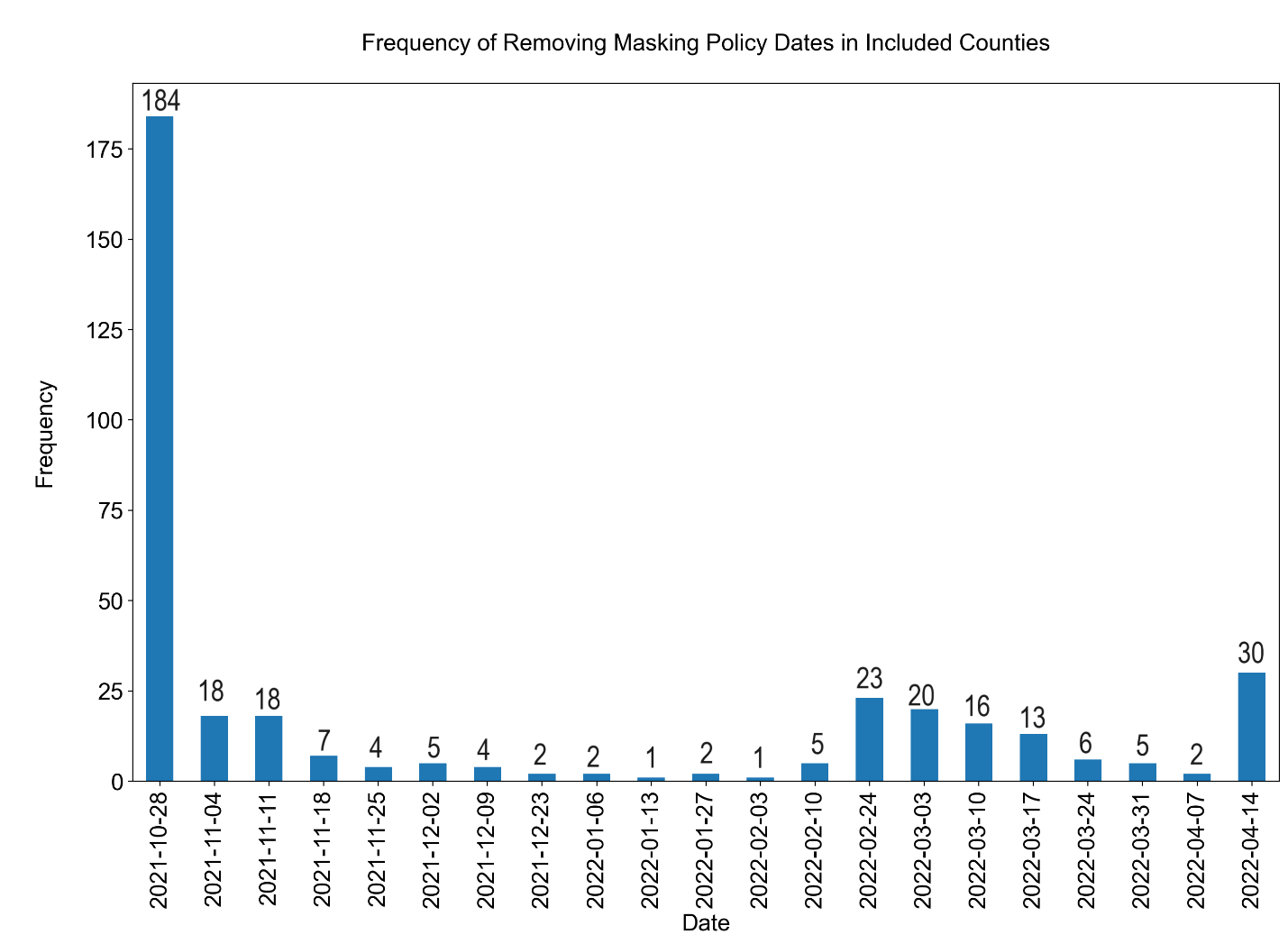


**Supplementary Materials**: Code underlying the analyses

clear

ssc install eventdd

ssc install matsort

ssc install xtitsa

ssc install reghdfe

import delimited "..\masking_data.csv"

gen date1= date(date, "YMD")

gen date2= date(no_policy_first_date, "YMD")

gen weeks_from_policy_change= round((date1 - date2)/7)

egen panel_var= group(school districtnces fips age_group student_asymp_testing)

xtset panel_var weeks_from_policy_change

gen cases_100k= (cases_cumsum/county_population) * 100000

gen hosp_1m= (hosp_cumsum/county_population) * 1000000

gen death_1m= (death_cumsum/county_population) * 1000000

**Generate Regions

generate region = "Northeast" if inlist(stateabbrev, "PA", "NY", "VT", "NH", "NJ", "MA", "CT", "RI", "ME")

replace region = "West" if inlist(stateabbrev, "AK", "WA", "OR", "CA", "ID")

replace region = "West" if inlist(stateabbrev,"NV", "MT", "WY", "UT", "AZ", "CO", "NM")

replace region = "Midwest" if inlist(stateabbrev, "ND", "SD", "NE", "KS", "MN", "IA")

replace region = "Midwest" if inlist(stateabbrev,"MO", "WI", "IL", "IN", "MI", "OH")

replace region = "South" if inlist(stateabbrev, "OK", "TX", "AR", "LA", "KY", "TN","NC", "SC")

replace region = "South" if inlist(stateabbrev,"MS", "AL", "GA", "FL", "WV", "MD", "DC", "VA")

gen student_testing= 0

replace student_testing= 1 if student_asymp_testing== "Unvaccinated_only"

replace student_testing= 2 if student_asymp_testing== "Partial"

replace student_testing= 3 if student_asymp_testing== "Selected_populations"

gen student_vaccine= 0

replace student_vaccine= 1 if student_vaccination== "High_school_limited"

replace student_vaccine= 2 if student_vaccination== "Eligible_populations"

gen one_week_lag = L1.weeks_from_policy_change

gen two_weeks_lag = L1.one_week_lag

*drop missing values caused by lags

drop if one_week_lag== .

drop if two_weeks_lag== .

egen avg_vax= rowmean(series_complete_511_pct series_complete_1217_pct series_complete_1864_pct series_complete_65_pct)

**##############################

**event study Cases - one weeks lag

**no age_group

eventdd cases_100k avg_vax month i.student_testing i.student_vaccine black_pcnt other_pcnt american_pcnt poverty_level grocery park transit workplace, accum baseline(0) lags(8) leads(4) timevar(one_week_lag) method(ols, cluster(panel_var)) graph_op(ytitle("Cases per 100,000") title("Cases per 100,000 County Population", size(medium) color(black)) xlabel(-4(1)8) xsize(80) ysize(60) scale(0.8))

*age 0-9

eventdd cases_100k series_complete_511 month i.student_testing i.student_vaccine black_pcnt other_pcnt american_pcnt poverty_level grocery park transit workplace if age_group== "0 - 9 Years" , accum baseline(0) lags(8) leads(4) timevar(one_week_lag) method(ols, cluster(panel_var)) graph_op(ytitle("Cases per 100,000") title("Cases per 100,000 County Population - Age Group 0-9", size(medium) color(black)) xlabel(-4(1)8) xsize(80) ysize(60) scale(0.8))

*age 10-19

eventdd cases_100k series_complete_1217 month i.student_testing i.student_vaccine black_pcnt other_pcnt american_pcnt poverty_level grocery park transit workplace if age_group== "10 - 19 Years", accum baseline(0) lags(8) leads(4) timevar(one_week_lag) method(ols, cluster(panel_var)) graph_op(ytitle("Cases per 100,000") title("Cases per 100,000 County Population - Age Group 10-19", size(medium) color(black)) xlabel(-4(1)8) xsize(80) ysize(60) scale(0.8))

*age 20-49

eventdd cases_100k series_complete_1864 month i.student_testing i.student_vaccine black_pcnt other_pcnt american_pcnt poverty_level grocery park transit workplace if age_group== "20 - 49 Years", accum baseline(0) lags(8) leads(4) timevar(one_week_lag) method(ols, cluster(panel_var)) graph_op(ytitle("Cases per 100,000") title("Cases per 100,000 County Population - Age Group 20-49", size(medium) color(black)) xlabel(-4(1)8) xsize(80) ysize(60) scale(0.8))

*age 50-69

eventdd cases_100k series_complete_1864 month i.student_testing i.student_vaccine black_pcnt other_pcnt american_pcnt poverty_level grocery park transit workplace if age_group== "50 - 69 Years", accum baseline(0) lags(8) leads(4) timevar(one_week_lag) method(ols, cluster(panel_var)) graph_op(ytitle("Cases per 100,000") title("Cases per 100,000 County Population - Age Group 50-69", size(medium) color(black)) xlabel(-4(1)8) xsize(80) ysize(60) scale(0.8))

*age 70+

eventdd cases_100k series_complete_65_pc month i.student_testing i.student_vaccine black_pcnt other_pcnt american_pcnt poverty_level grocery park transit workplace if age_group== "70+ Years", accum baseline(0) lags(8) leads(4) timevar(one_week_lag) method(ols, cluster(panel_var)) graph_op(ytitle("Cases per 100,000") title("Cases per 100,000 County Population - Age Group 70+", size(medium) color(black)) xlabel(-4(1)8) xsize(80) ysize(60) scale(0.8))

**event study Hospitalization - two weeks lag

**no age_group

eventdd hosp_1m avg_vax month i.student_testing i.student_vaccine black_pcnt other_pcnt american_pcnt poverty_level grocery park transit workplace, accum baseline(0) lags(8) leads(4) timevar(one_week_lag) method(ols, cluster(panel_var)) graph_op(ytitle("Hospitalization per 1,000,000") title("Hospitalization per 1,000,000 County Population", size(medium) color(black)) xlabel(-4(1)8) xsize(80) ysize(60) scale(0.8))

*age 0-9

eventdd hosp_1m series_complete_511 month i.student_testing i.student_vaccine black_pcnt other_pcnt american_pcnt poverty_level grocery park transit workplace if age_group== "0 - 9 Years", accum baseline(0) lags(8) leads(4) timevar(two_weeks_lag) method(ols, cluster(panel_var)) graph_op(ytitle("Hospitalization per 1,000,000") title("Hospitalization per 1,000,000 County Population - Age Group 0-9", size(medium) color(black)) xlabel(-4(1)8) xsize(80) ysize(60) scale(0.8))

*age 10-19

eventdd hosp_1m series_complete_1217 month i.student_testing i.student_vaccine black_pcnt other_pcnt american_pcnt poverty_level grocery park transit workplace if age_group== "10 - 19 Years", accum baseline(0) lags(8) leads(4) timevar(two_weeks_lag) method(ols, cluster(panel_var)) graph_op(ytitle("Hospitalization per 1,000,000") title("Hospitalization per 1,000,000 County Population - Age Group 10-19", size(medium) color(black)) xlabel(-4(1)8) xsize(80) ysize(60) scale(0.8))

*age 20-49

eventdd hosp_1m series_complete_1864 month i.student_testing i.student_vaccine black_pcnt other_pcnt american_pcnt poverty_level grocery park transit workplace if age_group== "20 - 49 Years", accum baseline(0) lags(8) leads(4) timevar(two_weeks_lag) method(ols, cluster(panel_var)) graph_op(ytitle("Hospitalization per 1,000,000") title("Hospitalization per 1,000,000 County Population - Age Group 20-49", size(medium) color(black)) xlabel(-4(1)8) xsize(80) ysize(60) scale(0.8))

*age 50-69

eventdd hosp_1m series_complete_1864 month i.student_testing i.student_vaccine black_pcnt other_pcnt american_pcnt poverty_level grocery park transit workplace if age_group== "50 - 69 Years", accum baseline(0) lags(8) leads(4) timevar(two_weeks_lag) method(ols, cluster(panel_var)) graph_op(ytitle("Hospitalization per 1,000,000") title("Hospitalization per 1,000,000 County Population - Age Group 50-69", size(medium) color(black)) xlabel(-4(1)8) xsize(80) ysize(60) scale(0.8))

*age 70+

eventdd hosp_1m series_complete_65_pc month i.student_testing i.student_vaccine black_pcnt other_pcnt american_pcnt poverty_level grocery park transit workplace if age_group== "70+ Years", accum baseline(0) lags(8) leads(4) timevar(two_weeks_lag) method(ols, cluster(panel_var)) graph_op(ytitle("Hospitalization per 1,000,000") title("Hospitalization per 1,000,000 County Population - Age Group 70+", size(medium) color(black)) xlabel(-4(1)8) xsize(80) ysize(60) scale(0.8))

**event study Death - two weeks lag

*no age_group

eventdd deaths_1m avg_vax month i.student_testing i.student_vaccine black_pcnt other_pcnt american_pcnt poverty_level grocery park transit workplace, accum baseline(0) lags(8) leads(4) timevar(one_week_lag) method(ols, cluster(panel_var)) graph_op(ytitle("Deaths per 1,000,000") title("Deaths per 1,000,000 County Population", size(medium) color(black)) xlabel(-4(1)8) xsize(80) ysize(60) scale(0.8))

*age 0-9

eventdd deaths_1m series_complete_511 month i.student_testing i.student_vaccine black_pcnt other_pcnt american_pcnt poverty_level grocery park transit workplace if age_group== "0 - 9 Years", accum baseline(0) lags(8) leads(4) timevar(two_weeks_lag) method(ols, cluster(panel_var)) graph_op(ytitle("Deaths per 1,000,000") title("Deaths per 1,000,000 County Population - Age Group 0-9", size(medium) color(black)) xlabel(-4(1)8) xsize(80) ysize(60) scale(0.8))

*age 10-19

eventdd deaths_1m series_complete_1217 month i.student_testing i.student_vaccine black_pcnt other_pcnt american_pcnt poverty_level grocery park transit workplace if age_group== "10 - 19 Years", accum baseline(0) lags(8) leads(4) timevar(two_weeks_lag) method(ols, cluster(panel_var)) graph_op(ytitle("Deaths per 1,000,000") title("Deaths per 1,000,000 County Population - Age Group 10-19", size(medium) color(black)) xlabel(-4(1)8) xsize(80) ysize(60) scale(0.8))

*age 20-49

eventdd deaths_1m series_complete_1864 month i.student_testing i.student_vaccine black_pcnt other_pcnt american_pcnt poverty_level grocery park transit workplace if age_group== "20 - 49 Years", accum baseline(0) lags(8) leads(4) timevar(two_weeks_lag) method(ols, cluster(panel_var)) graph_op(ytitle("Deaths per 1,000,000") title("Deaths per 1,000,000 County Population - Age Group 20-49", size(medium) color(black)) xlabel(-4(1)8) xsize(80) ysize(60) scale(0.8))

*age 50-69

eventdd deaths_1m series_complete_1864 month i.student_testing i.student_vaccine black_pcnt other_pcnt american_pcnt poverty_level grocery park transit workplace if age_group== "50 - 69 Years", accum baseline(0) lags(8) leads(4) timevar(two_weeks_lag) method(ols, cluster(panel_var)) graph_op(ytitle("Deaths per 1,000,000") title("Deaths per 1,000,000 County Population - Age Group 50-69", size(medium) color(black)) xlabel(-4(1)8) xsize(80) ysize(60) scale(0.8))

*age 70+

eventdd deaths_1m series_complete_65_pc month i.student_testing i.student_vaccine black_pcnt other_pcnt american_pcnt poverty_level grocery park transit workplace if age_group== "70+ Years", accum baseline(0) lags(8) leads(4) timevar(two_weeks_lag) method(ols, cluster(panel_var)) graph_op(ytitle("Deaths per 1,000,000") title("Deaths per 1,000,000 County Population - Age Group 70+", size(medium) color(black)) xlabel(-4(1)8) xsize(80) ysize(60) scale(0.8))

********************************************************************************

********************************************************************************

**Target Trial Emulation - IPW

clear

import delimited "..\target_trial_emulation_data.csv"

**Generate Regions

generate region = "Northeast" if inlist(stateabbrev, "PA", "NY", "VT", "NH", "NJ", "MA", "CT", "RI", "ME")

replace region = "West" if inlist(stateabbrev, "AK", "WA", "OR", "CA", "ID")

replace region = "West" if inlist(stateabbrev,"NV", "MT", "WY", "UT", "AZ", "CO", "NM")

replace region = "Midwest" if inlist(stateabbrev, "ND", "SD", "NE", "KS", "MN", "IA")

replace region = "Midwest" if inlist(stateabbrev,"MO", "WI", "IL", "IN", "MI", "OH")

replace region = "South" if inlist(stateabbrev, "OK", "TX", "AR", "LA", "KY", "TN","NC", "SC")

replace region = "South" if inlist(stateabbrev,"MS", "AL", "GA", "FL", "WV", "MD", "DC", "VA")

encode age_group, gen(age_group_enc)

encode region, gen(region_enc)

encode student_vaccination, gen(student_vaccination_enc)

encode student_asymp_testing, gen(student_asymp_testing_enc)

gen student_testing= 0

replace student_testing= 1 if student_asymp_testing== "Unvaccinated_only"

replace student_testing= 2 if student_asymp_testing== "Partial"

replace student_testing= 3 if student_asymp_testing== "Selected_populations"

gen student_vaccine= 0

replace student_vaccine= 1 if student_vaccination== "High_school_limited"

replace student_vaccine= 2 if student_vaccination== "Eligible_populations"

gen hosp_1m= (hops_100k) * 10

gen deaths_1m= (total_death/county_population) * 1000000

gen date1= date(date, "YMD")

egen avg_vax= rowmean(series_complete_511_pct series_complete_1217_pct series_complete_1864_pct series_complete_65_pct)

*removing first switch is control group

drop if fips== 1003 & date1>= date("2021-12-03", "YMD") & group== 0

drop if fips== 1103 & date1>= date("2021-12-24", "YMD") & group== 0

drop if fips== 21009 & date1>= date("2021-11-12", "YMD") & group== 0

drop if fips== 21085 & date1>= date("2021-12-03", "YMD") & group== 0

drop if fips== 21093 & date1>= date("2021-12-10", "YMD") & group== 0

drop if fips== 21207 & date1>= date("2021-11-26", "YMD") & group== 0

drop if fips== 30023 & date1>= date("2022-02-25", "YMD") & group== 0

drop if fips== 30035 & date1>= date("2021-12-17", "YMD") & group== 0

drop if fips== 30061 & date1>= date("2021-12-24", "YMD") & group== 0

drop if fips== 39131 & date1>= date("2022-02-25", "YMD") & group== 0

drop if fips== 48025 & date1>= date("2021-11-12", "YMD") & group== 0

drop if fips== 48249 & date1>= date("2022-05-06", "YMD") & group== 0

*removing first switch is treatment group

drop if fips== 13089 & date1>= date("2021-10-29", "YMD") & group== 1

drop if fips== 21041 & date1>= date("2021-10-29", "YMD") & group== 1

drop if fips== 21067 & date1>= date("2021-10-29", "YMD") & group== 1

drop if fips== 47037 & date1>= date("2021-10-29", "YMD") & group== 1

drop if fips== 39077 & date1>= date("2021-11-12", "YMD") & group== 1

**Whole Cohort

teffects ipw (cases_100k) (group date1 region_enc urban_int avg_vax risk_level grocery park residential retail transit workplace student_testing student_vaccine), pomeans

**Age stratified

teffects ipw (cases_100k) (group date1 region_enc urban_int series_complete_511_pct risk_level grocery park residential retail transit workplace student_testing student_vaccine) if age_group== "0 - 9 Years", pomeans

teffects ipw (cases_100k) (group date1 region_enc urban_int series_complete_1217_pct risk_level grocery park residential retail transit workplace student_testing student_vaccine) if age_group== "10 - 19 Years", pomeans

teffects ipw (cases_100k) (group date1 region_enc urban_int series_complete_1864_pct risk_level grocery park residential retail transit workplace student_testing student_vaccine) if age_group== "20 - 49 Years", pomeans

teffects ipw (cases_100k) (group date1 region_enc urban_int series_complete_1864_pct risk_level grocery park residential retail transit workplace student_testing student_vaccine) if age_group== "50 - 69 Years", pomeans

teffects ipw (cases_100k) (group date1 region_enc urban_int series_complete_65_pct risk_level grocery park residential retail transit workplace student_testing student_vaccine) if age_group== "70+ Years", pomeans

**********************************************************************

**Unadjusted**

*Cases

**Whole Cohort

teffects ipw (cases_100k) (group date1 region_enc urban_int), pomeans

**Age stratified

teffects ipw (cases_100k) (group date1 region_enc urban_int) if age_group== "0 - 9 Years", pomeans

teffects ipw (cases_100k) (group date1 region_enc urban_int) if age_group== "10 - 19 Years", pomeans

teffects ipw (cases_100k) (group date1 region_enc urban_int) if age_group== "20 - 50 Years", pomeans

teffects ipw (cases_100k) (group date1 region_enc urban_int) if age_group== "50 - 70 Years", pomeans

teffects ipw (cases_100k) (group date1 region_enc urban_int) if age_group== "70+ Years", pomeans

***********************************

*Hospitalizations

**Whole Cohort

teffects ipw (hosp_1m) (group date1 region_enc urban_int), pomeans

**Age stratified

teffects ipw (hosp_1m) (group date1 region_enc urban_int) if age_group== "0 - 9 Years", pomeans

teffects ipw (hosp_1m) (group date1 region_enc urban_int) if age_group== "10 - 19 Years", pomeans

teffects ipw (hosp_1m) (group date1 region_enc urban_int) if age_group== "20 - 50 Years", pomeans

teffects ipw (hosp_1m) (group date1 region_enc urban_int) if age_group== "50 - 70 Years", pomeans

teffects ipw (hosp_1m) (group date1 region_enc urban_int) if age_group== "70+ Years", pomeans

***********************************

*Deaths

**Whole Cohort

teffects ipw (deaths_1m) (group date1 region_enc urban_int), pomeans

**Age stratified

teffects ipw (deaths_1m) (group date1 region_enc urban_int) if age_group== "0 - 9 Years", pomeans

teffects ipw (deaths_1m) (group date1 region_enc urban_int) if age_group== "10 - 19 Years", pomeans

teffects ipw (deaths_1m) (group date1 region_enc urban_int) if age_group== "20 - 50 Years", pomeans

teffects ipw (deaths_1m) (group date1 region_enc urban_int) if age_group== "50 - 70 Years", pomeans

teffects ipw (deaths_1m) (group date1 region_enc urban_int) if age_group== "70+ Years", pomeans

************************************************
